# Metabolic engineering of a fast-growing cyanobacterium *Synechococcus elongatus* PCC 11801 for photoautotrophic production of succinic acid

**DOI:** 10.1186/s13068-020-01727-7

**Published:** 2020-05-18

**Authors:** Shinjinee Sengupta, Damini Jaiswal, Annesha Sengupta, Shikha Shah, Shruti Gadagkar, Pramod P. Wangikar

**Affiliations:** 1 Department of Chemical Engineering, Indian Institute of Technology Bombay, Powai, Mumbai, 400076 India; 2 DBT-Pan IIT Center for Bioenergy, Indian Institute of Technology Bombay, Powai, Mumbai, 400076 India; 3 Wadhwani Research Center for Bioengineering, Indian Institute of Technology Bombay, Powai, Mumbai, 400076 India

**Keywords:** Cyanobacteria, Succinic acid, Metabolites, Flux control

## Abstract

**Background:**

Cyanobacteria, a group of photosynthetic prokaryotes, are being increasingly explored for direct conversion of carbon dioxide to useful chemicals. However, efforts to engineer these photoautotrophs have resulted in low product titers. This may be ascribed to the bottlenecks in metabolic pathways, which need to be identified for rational engineering. We engineered the recently reported, fast-growing and robust cyanobacterium, *Synechococcus elongatus* PCC 11801 to produce succinate, an important platform chemical. Previously, engineering of the model cyanobacterium *S. elongatus* PCC 7942 has resulted in succinate titer of 0.43 g l^−1^ in 8 days.

**Results:**

Building on the previous report, expression of α-ketoglutarate decarboxylase, succinate semialdehyde dehydrogenase and phosphoenolpyruvate carboxylase yielded a succinate titer of 0.6 g l^−1^ in 5 days suggesting that PCC 11801 is better suited as host for production. Profiling of the engineered strains for 57 intermediate metabolites, a number of enzymes and qualitative analysis of key transcripts revealed potential flux control points. Based on this, we evaluated the effects of overexpression of sedoheptulose-1,7-bisphosphatase, citrate synthase and succinate transporters and knockout of succinate dehydrogenase and glycogen synthase A. The final construct with seven genes overexpressed and two genes knocked out resulted in photoautotrophic production of 0.93 g l^−1^ succinate in 5 days.

**Conclusion:**

While the fast-growing strain PCC 11801 yielded a much higher titer than the model strain, the efficient photoautotrophy of this novel isolate needs to be harnessed further for the production of desired chemicals. Engineered strains of *S. elongatus* PCC 11801 showed dramatic alterations in the levels of several metabolites suggesting far reaching effects of pathway engineering. Attempts to overexpress enzymes deemed to be flux controlling led to the emergence of other potential rate-limiting steps. Thus, this process of debottlenecking of the pathway needs to be repeated several times to obtain a significantly superior succinate titer.

## Background

Increasing concerns over the depletion of fossil fuels and the rise in the levels of atmospheric carbon dioxide have led to significant efforts toward a sustainable, bio-based economy. It is envisaged that biomass-derived fuels and commodity and specialty chemicals will replace those derived from petroleum-based products as the relevant technologies mature and become economically viable. As a roadmap in this direction, the US Department of Energy (DoE) has identified the top 15 platform chemicals along with the technology and research needs for their production from biomass [1]. Widely known as “DoE’s top-10 list”, the report provides valuable guidance to researchers and funding agencies working in the area of renewable chemicals. Succinic acid (SA) appears prominently in this list, both in the years 2004 and 2010 [1, 2]. This is due to (i) a strong succinic acid market of ~ 30,000 ton/year and (ii) a relatively mature technology for fermentative conversion of sugars to succinic acid that is getting commercialized [3]. Further, easy conversion of SA to other products may result in an additional demand.

Biological processes for the conversion of sugars to SA have been commercialized by companies such as Bioamber, Myriant and Succinity [4]. The processes involve genetically engineered, heterotrophic organisms ranging from the natural producers such as *Mannheimia succiniciproducens*, originally isolated from the rumen [5], to the conventional workhorse such as *E. coli*. The bio-production of succinic acid is achieved via the tricarboxylic acid (TCA) cycle or its derivative. Majority of the natural succinate producers utilize the reductive branch of the TCA cycle under anaerobic conditions. In this pathway, phosphoenolpyruvate (PEP) is carboxylated to oxaloacetate, which is then reduced to succinate via malate and fumarate. Alternatively, studies have reported the overexpression of glyoxylate shunt [6], which cleaves isocitrate to succinate and glyoxylate. Glyoxylate then reacts with acetyl-CoA to produce malate and subsequently regenerates the oxaloacetate needed for citrate synthesis. A highly active anaplerotic pathway such as that catalyzed by PEP carboxylase (PEPC) or PEP carboxykinase (PEPCK) appears to be a common theme across the succinate producing strains [7–9]. The reported SA titers have been in the range of 60–100 g l^−1^, yield (on sugars) of 1.0 g g^−1^ and volumetric productivity of 1.0–2.5 g l^−1^ h^−1^.

While the narrative of bio-economy has been mostly around the conversion of biomass to chemicals, technologies for the direct, photosynthetic conversion of CO_2_ to useful chemicals would be game changing [10]. In this regard, cyanobacteria are emerging as important host organisms due to their ability to perform oxygenic photosynthesis, increasing availability of synthetic biology tools [11–14] and a detailed understanding of their metabolic pathways [15–18]. A number of studies have shown engineering of cyanobacteria for the production of chemicals such as ethanol, *n*-butanol, 1,3-butanediol, isoprene, ethylene, isobutanol and succinic acid, although the reported titers have been low [19–21]. Majority of these studies have been with model cyanobacteria such as *Synechocystis* sp. PCC 6803 and *Synechococcus elongatus* PCC 7942, which suffer from disadvantages such as slower growth rates and lack of tolerance to high light and temperature and other abiotic stresses. It has been argued that significant improvements in the productivity can be achieved by employing fast-growing and stress-tolerant cyanobacteria such as *S. elongatus* UTEX 2973 [22, 23], PCC 11801 [24] and PCC 11802 [25].

The TCA cycle was thought to be incomplete in cyanobacteria due to the absence of 2-oxoglutarate dehydrogenase which converts α-ketoglutarate (α-KG) to succinyl coenzyme A [26]. This hypothesis has changed significantly after the discovery of a shunt in *Synechococcus* sp. PCC 7002 that converts α-KG to succinate [27]. The shunt operates by first converting α-KG to succinyl semialdehyde by α-ketoglutarate decarboxylase (OgdA) and then to succinate by succinyl semialdehyde dehydrogenase (SsaD). Though the flux through this branch of the TCA cycle is reported to be low under photoautotrophic conditions, this pathway was then engineered in cyanobacterium *S. elongatus* PCC 7942 along with citrate synthase (*gltA*) and the anaplerotic pathway of *PEPC*, all under the control of a single inducible promoter P_trc_ [28]. This pioneering study on photosynthetic conversion of CO_2_ to succinate reported the highest product titer of 430 mg l^−1^ in 8 days. Other more recent studies have not reported any significant improvement over this titer under photoautotrophic growth [29]. A recent study reports dark fermentative production of succinate from photo-autotrophically grown cyanobacteria at titer of 1.8 g l^−1^ [30, 31]. However, this study used very high biomass concentration that was grown in dilute photoautotrophic cultures, thus amounting to a very low specific and volumetric productivity.

Building on the previous reports, we have engineered a fast-growing cyanobacterium *S. elongatus* PCC 11801 to obtain a significantly higher succinate titer under photoautotrophic growth. Our approach involves the identification of flux control points via metabolic profiling of the wild-type and engineered strains. The key rate-limiting enzyme of sedoheptulose bisphosphatase (*SBPase*) [32] was overexpressed while the glycogen synthase A (*glgA*) [33] and succinate dehydrogenase subunit B (*sdhB*) [34] was knocked out to achieve further improvements in the titer. The recently reported succinate transporter genes *yjjPB* of *E. coli* were overexpressed to alleviate the intracellular accumulation of the product. Our results provide useful insights into the metabolism of this fast-growing cyanobacterium that may be of interest to the broader metabolic engineering community.

## Results

Cyanobacterium *S. elongatus* PCC 11801 was selected as the host strain because of its fast growth rate and tolerance to various abiotic stresses such as high temperature, light and CO_2_ [24]. Higher growth rates, which may result from higher rates of photosynthesis and carbon fixation, can potentially be translated into higher product formation rates via metabolic engineering. As a first step, we assessed the tolerance of *S. elongatus* PCC 11801 toward the product of interest, succinic acid. Toward this, the strain was grown in BG-11 medium containing disodium succinate in the concentration range of 0–500 mM. No appreciable growth retardation was observed up to 125 mM (Additional file 1: Figure S1a). Growth was also monitored under equivalent concentrations of NaCl to assess if the growth inhibition was due to salt stress. Notably, the growth retardation observed at 250 and 500 mM succinate was also observed in the culture containing twice the concentration of NaCl. PCC 11801 tolerated higher levels of succinate compared with *S. elongatus* PCC 7942 [28] (Additional file 1: Figure S1a). Further, *S. elongatus* PCC 11801 did not consume succinic acid upon incubation with 1 g l^−1^ succinate under photoautotrophic growth conditions (Additional file 1: Figure S1b).

### Strain engineering strategy

**Fig. 1:**
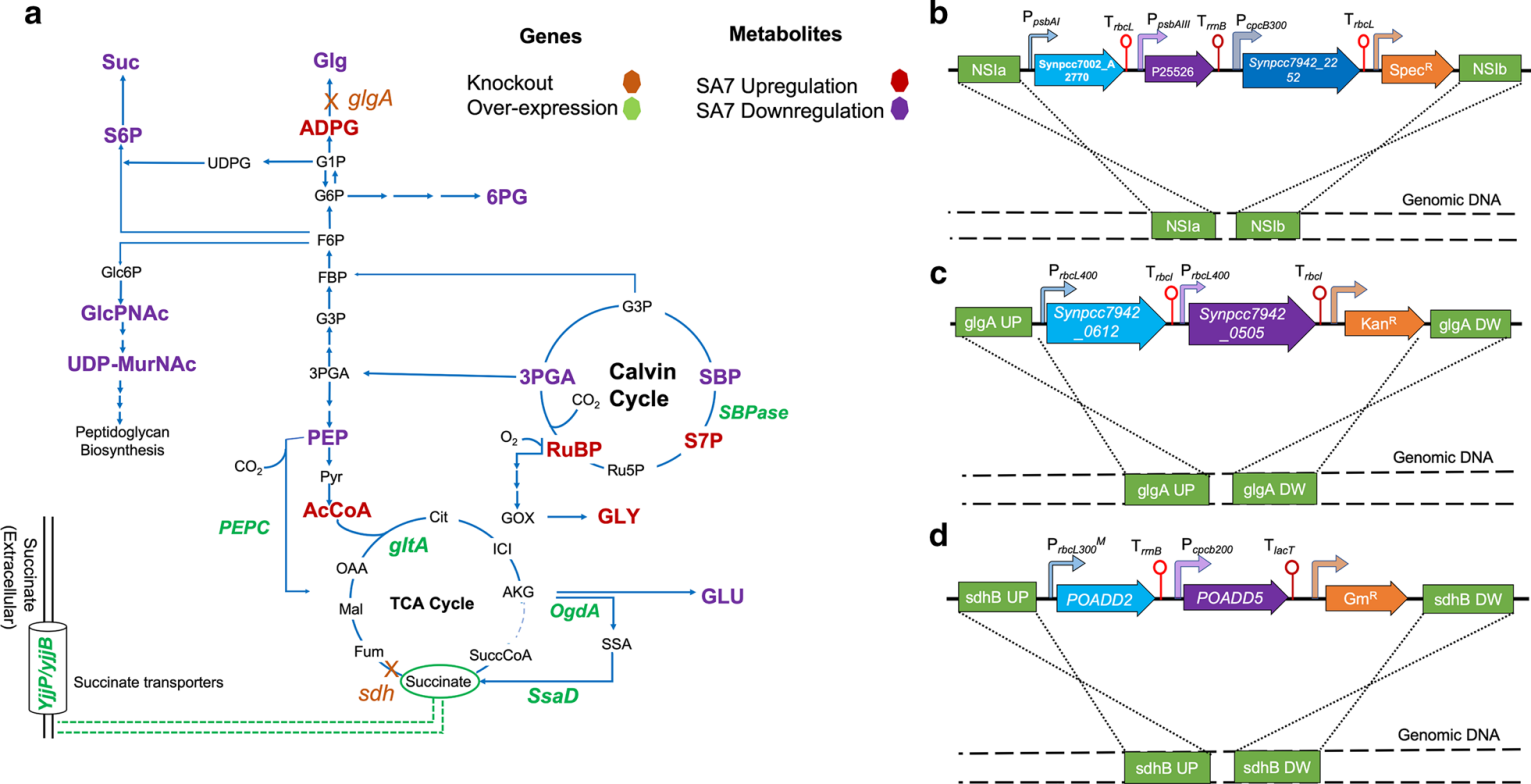
Overview of the pathway engineering strategy for succinate production in *S. elongatus* PCC 11801. **a** An abridged central carbon pathway with the overexpressed genes shown in green font and genes that are knocked out in orange font with a cross on the reaction arrow. Blue lines represent the native pathway of PCC 11801. Different subsets of genetic modifications were incorporated in strains SA1–SA8 while all the modifications are present in SA9. Strain details in Table 1 and Additional file 1: Table S1. Results of metabolomics studies for the strain SA7 are superimposed on the pathway by color coding the metabolites. The metabolites depicted in purple show depletion while the ones in red show accumulation in *S. elongatus* PCC 11801 SA7 compared to the wild-type. **b** Schematic for homologous recombination of the three gene construct into the NSI site of *S. elongatus* PCC 11801 chromosome. The locus tag are: *OgdA*-Synpcc7002_A2770, *SsaD*-P25526, and *PEPC*-Synpcc7942_2252. **c** Schematic showing homologous recombination of a construct of Synpcc7942_0612 (*gltA*), Synpcc7942_0505 (*SBPase*) at the *glgA* site. **d** Schematic showing homologous recombination of *E. coli* succinate transporter genes, POADD2 (*yjjP*) and POADD5 (*yjjB*) at the *sdhB* site of PCC 11801. *Enzymes*: PEPC, PEP carboxylase; GltA, citrate synthase; OgdA, 2-ketoglutarate decarboxylase; SsaD, NADP-dependent succinate semialdehyde dehydrogenase, sdhB, Succinate dehydrogenase subunit B; SBPase/FBPase, fructose-1,6-biphosphatase/sedoheptulose-1,7-biphosphatase; glgA, glycogen synthase; *metabolites*: Glg, glycogen; ADPG, adenosine-5′-diphosphoglucose; G1P, glucose 1-phosphate; G6P, glucose 6-phosphate; F6P, fructose 6-phosphate; FBP, fructose bisphosphatase; G3P, glyceraldehyde 3-phosphate; 3PGA, 3-phosphoglyceric acid; PEP, phosphoenolpyruvate; Pyr, pyruvate; AcCoA, acetyl CoA; Cit, citrate; ICI, isocitrate; α-KG, alpha ketoglutarate; SuccCoA, succinyl CoA; Fum, fumarate; Mal, malate; OAA, oxaloacetate; RuBP, ribulose 1,5-bisphosphate; Ru5P, ribose 5-phosphate; S7P, sedoheptulose 7-phosphate; SBP, sedoheptulose 1,7-bisphosphate; GOX, glycolate oxidase; GLY, glycine; GLU, glutamate; 6PG, 6-phosphogluconate; Suc, sucrose; Suc6P, sucrose 6-phosphate; Glc6P, glucose-6-phosphate; GlcPNAc, *N*-acetylglucosamine; UDP-MurNAc, UDP-*N*-acetylmuramic acid. Additional details of the metabolites in Additional file 1: Table S6

The overall metabolic engineering strategy adopted in this study is summarized in Fig. 1a. The recombinant strains constructed in this study are listed in Table 1 with additional details in Additional file 1: Table S1.

**Table 1:** List of the recombinant strains constructed in this study

Strain	∆ NSI (∆DOP62_03525)^a^				∆glgA (∆DOP62_03790)^a^			∆ sdhB (∆DOP62_11515)^a^		
	Genes overexpressed^b^/knocked out									
	*NSI*	*OgdA*	*SsaD*	*PEPC*	*glgA*	*gltA*	*SBPase*	*sdhB*	*YjjP*	*YjjB*
WT	–	–	–	–	–	–	–	–	–	–
SA1	X	✓	✓	–	–	–	–	–	–	–
SA2	X	✓	✓	–	–	–	–	–	–	–
SA3	X	✓	✓	✓	–	–	–	–	–	–
SA4	X	✓	✓	✓	–	–	–	X	–	–
SA5	X	✓	✓	✓	X	–	–	–	–	–
SA6	X	✓	✓	✓	X	✓	–	–	–	–
SA7	X	✓	✓	✓	X	✓	✓	–	–	–
SA8	X	✓	✓	✓	X	✓	✓	X	–	–
SA9	X	✓	✓	✓	X	✓	✓	X	✓	✓

Strains SA1-SA3 were constructed in *S. elongatus* PCC 7942 and PCC 11801 while the strains SA4-SA9 were constructed in PCC 11801 alone

^a^Locus tags of the sites where constructs are integrated

^b^The locus tag/Uniprot identifiers as follows *OgdA* (Synpcc7002_A2770), *SsaD* (P25526), *PEPC* (Synpcc7942_2252), *gltA* (Synpcc7942_0612), *Sbpase (*Synpcc7942_0505), *yjjP* (POADD2) and *yjjB* (POADD5)

X—knockout/disrupted site

✓—genes overexpressed

The genes overexpressed in PCC 11801 were chromosomally integrated along with antibiotic markers [35] at one of the following sites (gene name, locus tag): neutral site I (NS-I), DOP62_03525; *glgA*, DOP62_03790; and *sdhB*, DOP62_11515 (Additional file 1: Table S1, Fig. 1b–d). First, the relevant plasmids were constructed (Additional file 1: Table S2), which were then transformed into *S. elongatus* PCC 11801 or PCC 7942 to obtain the engineered strains SA1–SA9 (Additional file 1: Table S3). Primers used in this study are listed in Additional file 1: Table S4. Complete chromosomal segregation was achieved for each of the recombinant strains (Additional file 1: Figure S2). While NS-I of PCC 11801 shares ~ 83% identity with that of PCC 7942 [24], integration at the other two sites are expected to result in disruption of the respective gene. As a general strategy, each gene was expressed under the control of a separate promoter with up to 3 genes integrated at each of the chromosomal sites. Native promoters of PCC 7942 were used as they have been well-characterized previously [12, 36]. Further, the respective promoter sequences of *S. elongatus* PCC 11801 and PCC 7942 share ~ 83% identity. The DNA fragments were assembled by overlap extension PCR and cloned into an appropriate vector. The vectors pSYN1 and pSYN6 were used for homologous recombination at the NS-I site (Fig. 1b) while the vectors pAM1619 and pAM3558 with modified homology arms were used for integration at the sites *glgA* and *sdhB*, respectively (Fig. 1c, d). As a first step, the two genes reported for the succinate pathway, *OgdA* and *SsaD* were overexpressed under the control of strong promoters from PCC 7942, P_*rbcL400*_ and P_*psbaIII*_, respectively, to obtain PCC 11801 SA1. For SA2, P_*psbaI*_ was chosen in place of P_*rbcL400*_ as the former is known to be repressed under high light [37] and may help avoid the buildup of the toxic intermediate succinic semialdehyde. The genes *OgdA* and *SsaD* were selected from *Synechococcus* sp. PCC 7002 and *E. coli*, respectively, based on previous reports [27, 28]. Overexpression of these genes in PCC 7942 has been shown to result in growth retardation, possibly due to the depletion of TCA cycle intermediates, a phenotype that was rescued by overexpression of *PEPC* [28]. Overexpression of *PEPC* has been widely reported as a strategy for improving succinate production [28]. Thus, the strain SA2 was modified by including a cassette for overexpression of the gene *PEPC* from *S. elongatus* PCC 7942, under the control of a super-strong native truncated promoter P_*cpcb300*_ [12] to obtain PCC 11801 SA3 (Fig. 1b, Table 1). Likewise, equivalent strains PCC 7942 SA1–SA3 were constructed for direct comparison of succinate titers.

After establishing superior succinate productivity in PCC 11801, the strains SA4–SA9 were then constructed only in PCC 11801 (Table 1). The design of these strains was based on the identification of flux control points via metabolite profiling, activity assays of selected enzymes and extracellular accumulation of fumarate. The gene *sdhB* was knocked out to prevent the conversion of succinate to fumarate to obtain the strain SA4. Further, metabolite profiles of strains SA2 and SA3 showed significant accumulation of ADP-glucose, a precursor of glycogen synthesis, suggesting down-regulation of *glgA*. This appears to be a consequence of channeling of carbon flux toward succinate and a part of the organism’s strategy of metabolic adjustment. Moreover, knockout of glycogen synthesis has been reported as a metabolic engineering strategy to improve product titer by creating a condition referred to as metabolic overflow [38]. The glycogen biosynthetic pathway of *Synechocystis* sp. PCC 6803 comprises ADP-glucose pyrophosphorylase (AGPase coded by *glgC*), glycogen synthase genes *glgA1* and *glgA2* and the branching enzyme *glgB*. The mutant strains of cyanobacteria cannot produce detectable amounts of glycogen if the *glgC* or both *glgA1* and *glgA2* are knocked out [39–41]. However, in *S. elongatus* PCC 11801, only one copy of *glgA* is present. Thus, the gene *glgA* was knocked out to obtain strains SA5 and SA7 (Additional file 1: Figure S3). The gene *glgC* was not chosen as a target for knockout as that would have abolished the production of glucosylglycerol as well. Knockout of *glgA* resulted in 50% reduction in glycogen content in SA7 (Fig. 5) suggesting that PCC 11801 may be able to synthesize glycogen via some other enzymes as reported in other organisms [42]. As both SA4 and SA5 showed improvement in the succinate titer, the subsequent genes were overexpressed at one of these loci. The enzyme citrate synthase (*gltA*) is the first committing step of the TCA cycle and therefore is likely to be a potential flux controlling step. Indeed, *gltA* was found to be down-regulated in SA3 based on the enzyme activity measurement. Therefore, *gltA* was chosen as the next target gene chosen for overexpression. Further, the metabolite profiles of SA2 and SA3 show the accumulation of sedoheptulose-1,7-bisphosphate (SBP) and depletion of sedoheptulose-7-phosphate (S7P). These are the substrate and product, respectively, of the enzyme SBPase, suggesting that this may be another flux control point in these engineered strains. See additional details in “Metabolite profiling to identify bottlenecks” section. Thus, overexpression of *SBPase* was included in the strain design strategy to improve the carbon fixation efficiency. Next, we observed a twofold accumulation of intracellular succinate in SA7 suggesting that the secretion of this product may be one of the limiting steps. Therefore, the *E. coli* genes encoding for succinate transporter, *yjjPB*, were overexpressed under the control of low strength promoters, a truncated native promotor P_*cpcb200*_ [12] and a mutant of P_rbc300_ (A. Sengupta et al., unpublished results). Promoters of lower strengths were selected for these membrane proteins as has been recommended in previous reports [43]. This is primarily to avoid aggregation of the protein and also to maintain the membrane integrity by avoiding overloading of the membrane with the heterologous protein. The genes *yjjPB* have been reported to enhance succinate production in *Pantoea ananatis* under aerobic conditions [44]. Note that succinate transporters of cyanobacterial origin have not yet been reported.

### Characterization of the engineered strains

#### Growth profiles

**Fig. 2:**
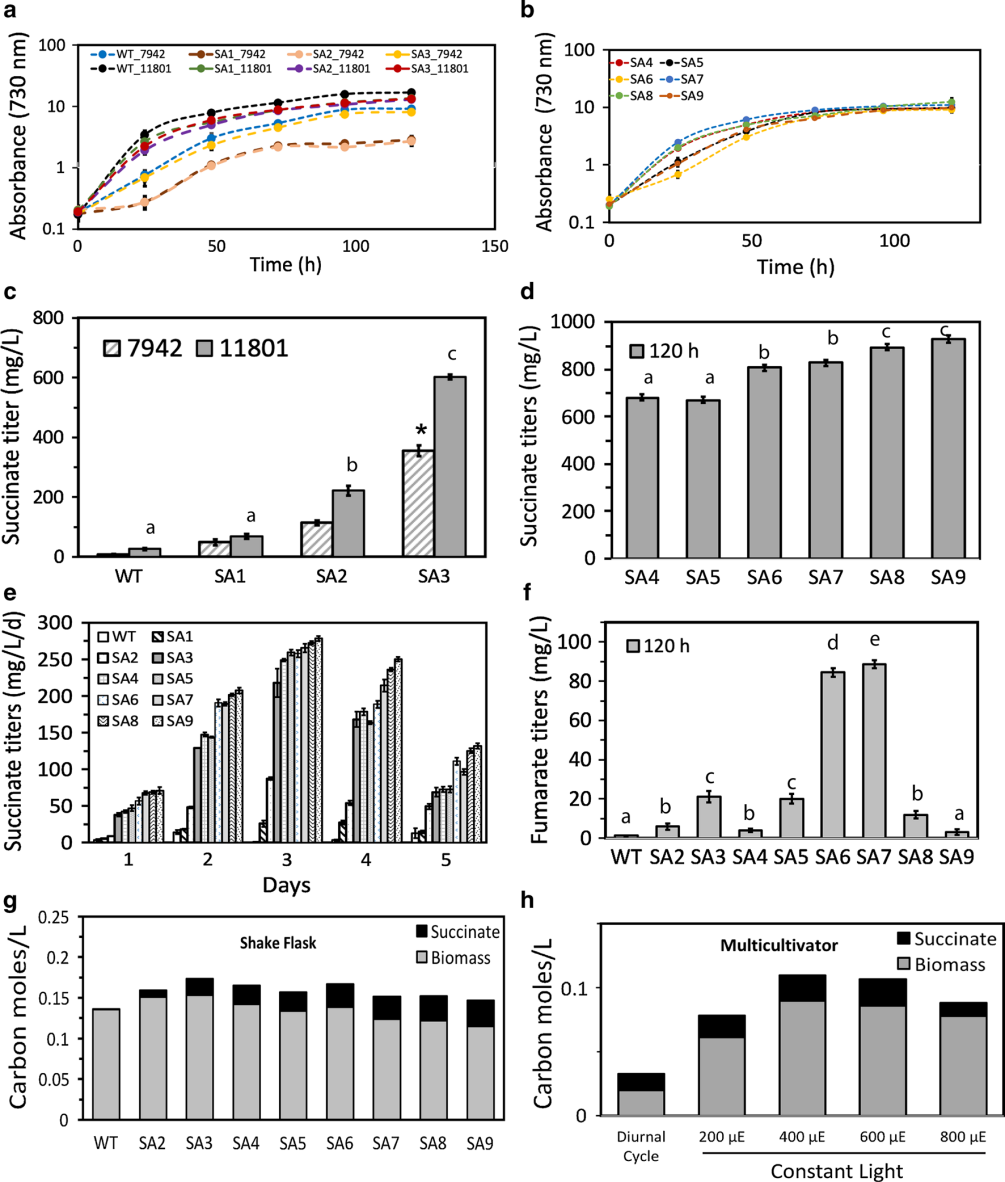
Characterization of succinate producing strains of *S. elongatus*. **a** Growth profiles of strains WT and SA1–SA3 of PCC 7942 and PCC 11801 grown in BG-11 media in under 1% CO_2_ in a shaker. **b** Growth profiles of strains *S. elongatus* PCC 11801 SA4–SA9, **c** cumulative 120 h titers for strain SA1, SA2, SA3 and wild-type in *S. elongatus* PCC 11801 and 7942, **d** cumulative succinate titers at 120 h for strains SA4-SA9 of PCC 11801, **e** volumetric productivity of succinate of all the *S. elongatus* PCC 11801 succinate strains for 5 days. **f** Estimation of fumarate titers in all the *S. elongatus* PCC 11801 strains. **g** Estimation of carbon partitioning between succinate and biomass for engineered strains of *S. elongatus* PCC 11801. The strains were grown in shake flasks, at 1% CO_2_ in shaker, **h** estimation of carbon partitioning of *S. elongatus* PCC 11801 SA3 strain compared under different growth conditions. Diurnal cycle—light and dark condition in multicultivator, different light conditions 200, 400, 600 and 800 µE in multicultivator with 1% CO_2_ bubbling. Data shown above are mean of three independent experimental sets with error bars indicating standard error of the mean

Based on the previously reported growth conditions [24], we monitored the growth and succinate titer of the engineered *S. elongatus* PCC 11801 and PCC 7942 strains at 38 °C under 1% CO_2_ or ambient air with or without 50 mM NaHCO_3_ and light intensity of 300 µmole photons m^−2^ s^−1^. In general, the growth rates and 5-day biomass accumulation were in the order of 1% CO_2_ > 50 mM NaHCO_3_ > ambient air (Additional file 1: Figure S4a). Further, for the strain SA2, higher succinate titers were observed under 1% CO_2_ (Additional file 1: Figure S4b). Therefore, all subsequent trials were performed under 1% CO_2_. The engineered strains of PCC 11801 showed minor lag in growth in the first 24 h but the 5-day biomass accumulation was comparable to PCC 11801 wild-type strain (Fig. 2a, b).

Notably, the strains PCC 7942 SA1 and SA2 showed a marked growth defect compared to the wild-type PCC 7942, which was rescued in PCC 7942 SA3 (Fig. 2a). Similar growth retardation has been reported in *S. elongatus* PCC 7942 when *OgdA* and *SsaD* were expressed under inducible promoter, possibly due to depletion of the TCA cycle intermediates [28]. While minor lag in the growth was observed for strains PCC 11801 SA1 and SA2, the growth profile of SA3 was similar to the wild-type strain (Fig. 2a). On the other hand, SA6 displayed appreciable growth retardation possibly due to metabolic imbalance (Fig. 2b). This growth defect was rescued in the *SBPase* overexpressing strain SA7 (Fig. 2b). Refer to “Metabolic profiling to identify bottlenecks” section for the rationale behind choosing *SBPase* as the target for overexpression. The *sdhB* knockout strain SA8 and succinate transporter overexpressing strain SA9 resulted in longer lag phases but the 5-day culture OD_730_ was unaltered compared to the wild-type strain (Fig. 2b).

#### Succinate production

The overall progression of the product titer with the successive genetic modifications is shown in Fig. 2c, d. Among the two initial constructs, *S. elongatus* PCC 11801 SA2 showed a 5-day product titer of 250 mg l^−1^ when grown under 1% CO_2_ (Fig. 2c). Overexpression of *PEPC* in this strain resulted in significant improvement yielding succinate titer of 615 mg l^−1^ (Fig. 2c). The succinate titers obtained in the recombinant strains of PCC 7942, SA1–SA3, were significantly lower than the equivalent strains of PCC 11801 (Fig. 2c) suggesting that while the former may be suitable as a model organism for initial metabolic engineering studies, the latter may be more suitable as a host for production (Fig. 2c). To confirm the succinate production, end point determination of succinate was performed with GC–MS (Additional file 1: Figure S5). The *glgA* knockout strain *S. elongatus* PCC 11801 SA5 resulted in a marginally higher titer of ~ 685 mg l^−1^ probably due to low glycogen accumulation (Fig. 2d, e). Interestingly, another significant jump in the product titer was obtained with the overexpression of *gltA* resulting in a titer of 850 mg l^−1^ (Fig. 2d). Overexpression of *SBPase* in PCC 11801 SA7 resulted in improved growth but no significant change in the succinate titer (Fig. 2d). This is in agreement with previous reports of *SBPase* overexpression resulting in 15% higher total biomass in *Synechocystis* sp. PCC 6803 [32] or enhanced photosynthetic carbon capture in *Synechococcus* sp. PCC 7002 [45]. Next, it was of interest to minimize the undesirable byproducts and thus improve the succinate titer. As expected, the fumarate titers correlated well with the succinate titers in the strains SA2, SA3 and SA5–SA7 with the highest fumarate titers of ~ 90 mg l^−1^ observed in SA6 and SA7 (Fig. 2f). The *sdhB* gene knockout SA4 and SA8 superimposed on the strains SA3 and SA7, respectively, showed a decrease in the fumarate titers and concomitant increase in the succinate titers compared to the corresponding background strain. Finally, strain SA9 with succinate transporters from *E. coli* overexpressed in SA8 showed the highest titer of ~ 930 mg l^−1^. Interestingly, the fumarate titer was the lowest in SA9. It is of interest to maintain high volumetric succinate productivity for several days of a batch culture in order to obtain a high titer. It was observed that the productivity peaks on the third day and then declines for all the strains (Fig. 2e).

An ideal production strain should be able to partition a major proportion of the fixed carbon toward the product of interest. To that end, carbon partitioning between biomass and succinate in the engineered strains of PCC 11801 was estimated. The number of carbon moles in the biomass was computed from the dry cell weight and by assuming the elemental composition of CH_1.8_O_0.5_N_0.15_ [46, 47]. As expected, carbon partition towards the product increased with the successive engineered strains and was observed to be maximum for SA9 (Fig. 2g). Further, we studied the carbon partitioning under different conditions for the representative strain PCC 11801 SA3 (Fig. 2h). Under diurnal cycles, the relative carbon partitioning was higher than under other conditions tested. This maybe due to the fact that the flux through the TCA cycle is higher during the dark phase [48]. However, the highest succinate titer was observed under continuous light of 400 µE light and 1% CO_2_ although this condition also leads to higher biomass growth.

#### mRNA transcript levels

**Fig. 3:**
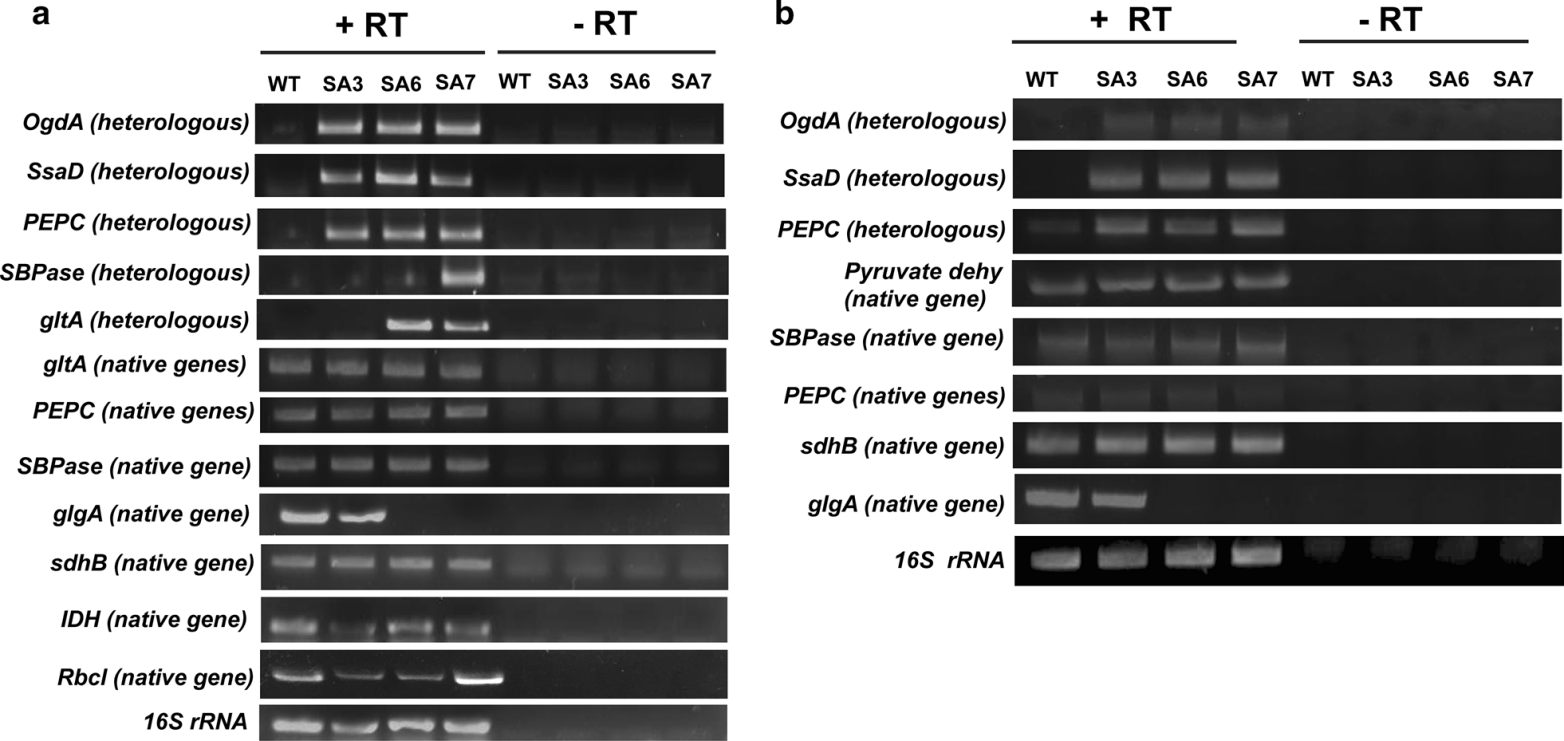
mRNA expression levels of the succinate pathway genes in selected strains. mRNA expression was observed in SA3, SA6 and SA7 strains in comparison to the wild-type for the exponential phase of growth (**a**) and late phase or stationary growth phase (**b**) of the cells. Primers amplifying approximately 200 bp amplicon were designed for each gene (Additional file 1: Table S5). RNA isolation and cDNA synthesis were performed by using commercially available kits and as per manufacturer’s protocols. The experiment was performed in duplicate and representative images of PCR products after gel electrophoresis are shown. 16S rRNA was used as a loading control. No reverse transcriptase control was treated as a negative control for each gene

The transcript levels of the heterologously expressed genes *OgdA*, *SsaD*, *PEPC*, *gltA* and *SBPase* and the native genes *PEPC*, *gltA*, *SBPase*, *glgA*, *sdhB*, *IDH* (isocitrate dehydrogenase) and *Rbcl* (RuBisCO) were assessed qualitatively via reverse transcriptase-PCR and gel electrophoresis for the engineered strains and under exponential growth phase using primers listed in Additional file 1: Table S5. Importantly, all the heterologously expressed genes showed satisfactory expression in the respective strains (Fig. 3a).

For *PEPC*, *gltA* and *SBPase*, the distinction between the native and heterologous genes was achieved by designing specific primers and the latter showed stronger expression as expected. Among the native genes, *sdhB* showed strong expression in all the strains (Fig. 3a). Further, *IDH* showed weak expression in SA3 while *Rbcl* showed lower transcript abundance in SA3 and SA6, which was restored to the wild-type levels in SA7. We believe that such gene repression may be a result of metabolic adjustment that the cell makes to maintain a certain flux distribution in the various branches of metabolism. Moreover, the genes *IDH* and *Rbcl* may be selected as targets for further engineering of the strain to improve succinate titer. Next, it was of interest to monitor if there was any decline in the expression of these genes in the late phase of growth (at OD_730_ ~ 10, *t* = 3 days) as the succinate production slows down beyond 3 days and stagnates beyond 5 days in all the strains. Expression of all the genes tested, both native and heterologous, was lower than that observed in the exponential growth phase (Fig. 3b). This may be an effect of the high cell density and slower growth in this phase. These results suggest that it may be imperative to design strains whose cell growth can be limited to low cell densities and in turn maintain an active metabolic state throughout the batch.

#### Enzyme assays

**Fig. 4:**
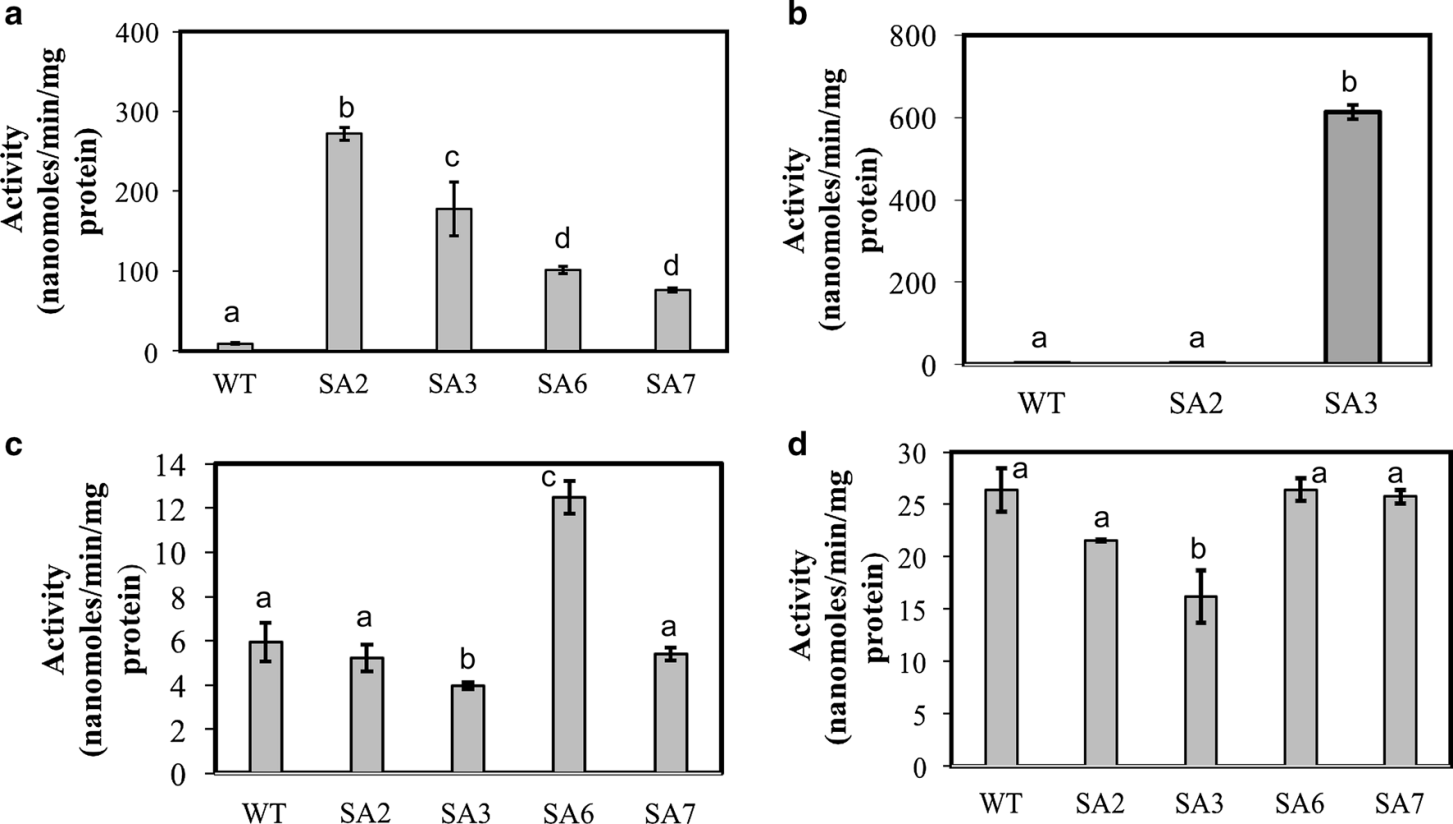
Enzymatic assay of key enzymes for the selected engineered strain. Enzymatic assay of **a** succinate semialdehyde dehydrogenase, SsaD, **b** PEP Carboxylase, PEPC **c** citrate synthase, GltA and **d** isocitrate dehydrogenase, IDH in the representative strains WT, SA2, SA3, SA6 and SA7 was monitored. The cells were harvested in the exponential phase of growth (OD_730_ ~ 0.8), centrifuged and lysed in a bead beater. Substrate blank was used as a control. Activity is measured in nanomoles/min/mg of crude protein in the lysate. Error bars represent standard error of mean of three trials

To assess the level of overexpression and identify the flux control points, in vitro enzyme assays were performed for the enzymes SsaD, PEPC, GltA, and IDH using crude extracts of selected engineered strains of PCC 11801 (Fig. 4, Additional file 1: Figure S6). Overexpression of *SsaD* in PCC 11801 SA2 resulted in over 100-fold increase in its activity compared to the WT strain (Fig. 4a). However, in strains SA3 and SA7, the *SsaD* activity decreased to ~ 60% and ~ 25% of that in SA2, respectively. Note that *SsaD* has been expressed under the constitutive promoter P_psbAIII_ in all the engineered strains and hence this variation in the activity may be a result of differences in the metabolic states of the different strains, which in turn may change the activity of this native, inducer-free promoter. Next, PEPC activity was found to be ~ 170-fold higher in the strain SA3 compared to SA2 (Fig. 4b), accompanied by a ~ 2.5-fold increase in the succinate titer. This suggests that PEPC may be a flux controlling step in SA2 but not in SA3. Interestingly, the activities of the enzymes GltA (Fig. 4c) and IDH (Fig. 4d) are lower in SA3 than those in the WT strain suggesting that one or both of these enzymes may act as flux controlling reactions in SA3. Overexpression of the *gltA* gene in SA6 results in enhanced activity of not only the GltA enzyme but also the IDH enzyme. Further, overexpression of SBPase in SA7 leads to decline of GltA activity suggesting that the later may be a key flux controlling enzyme that plays a role in regulating flux through the TCA cycle. In fact, repression of *gltA* has been successfully used as a strategy to limit the growth of cyanobacteria and improve product formation [49].

#### Metabolite profiling to identify bottlenecks

**Fig. 5:**
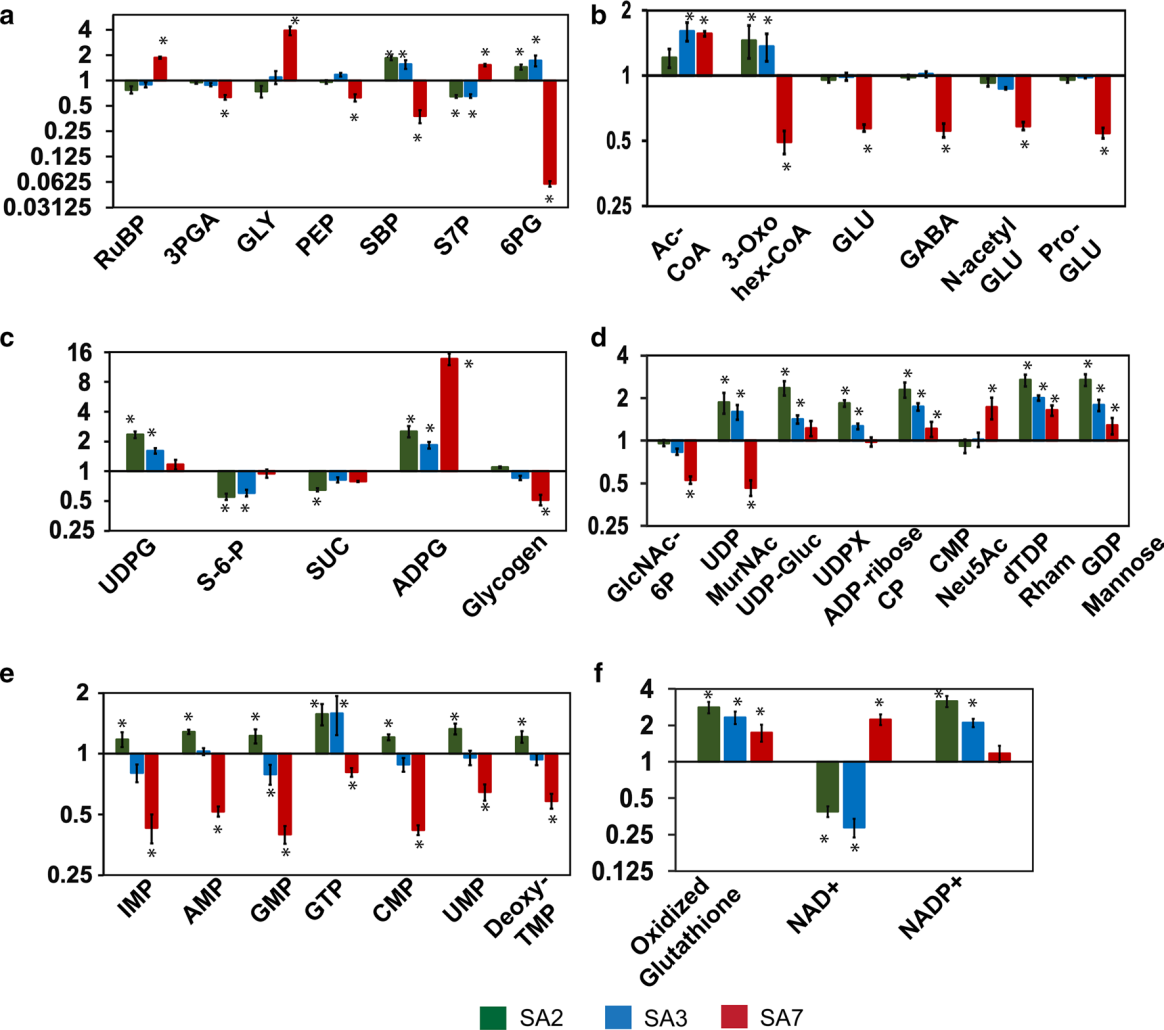
Metabolome profiling of the selected succinate producing strains of *S. elongatus* PCC 11801 in exponential growth phase. Fold change of the metabolite pool sizes with respect to the wild-type strain with data arranged according to major metabolic pathways or metabolite categories: **a** Calvin–Benson–Bassham (CBB) cycle; **b** TCA cycle and related metabolites. **c** Major storage molecules and their precursors, **d** nucleotide sugars, **e** nucleotides, **f** glutathione, NAD^+^ and NADP. Metabolite pool size in the engineered strains with respect to wild-type. All the data, barring glycogen, was collected using liquid chromatography coupled to high resolution mass spectrometry (HR-LC/MS). Cyanobacterial cultures were grown under 1% CO_2_ in a shaker and samples drawn in exponential growth phase (OD_730_ ~ 0.6) for metabolite extraction. The fully ^13^C labeled metabolite extract from PCC 11801 wild-type was used as the internal standard to use the isotopic ratio method of relative quantification. Further, ratio of pool size in the recombinant strain to that in the wild-type was obtained and The metabolites presented in this study were identified at MS2 level using the MS-DIAL and MetDIA tool [70, 71]. The data for metabolites with a fold change of > 1.5 or less than 0.66 and a *p*-value of < 0.05 using t-test in at least one of succinate producer strains are shown. Averages for three biological and three technical replicates (*n* = 9) are shown for clarity. Glycogen content was estimated as reported previously [75]. Details of the metabolites in Additional file 1: Table S6

It was of interest to assess the metabolic adjustments that the cell makes in response to the additional metabolic burden caused by the heterologously expressed pathway and in turn to identify the potential flux controlling steps. The pool sizes of 59 targeted metabolites comprising intermediates of central carbon metabolism, amino acids, nucleotides, nucleotide sugars, and cofactors were monitored in selected engineered and WT strains. The metabolite levels were quantified under the exponential growth phase by using the isotopic ratio method (Fig. 5, Additional file 1: Figures S7–S13) [50, 51] and expressed as fold change with respect to the levels in WT (For the complete list, see Additional file 1: Table S6). Metabolites with fold changes of greater than 1.5 and *p-* value of less than 0.05 are shown in Fig. 5.

We chose three of the engineered strains for the metabolomic study; SA2, SA3 and SA7. These strains have shown statistically significant improvement in succinate titer in the order SA7 > SA3 > SA2 > WT. The strains SA2 and SA3 showed significant accumulation of SBP and depletion of S7P, the reactant and product, respectively, of the enzyme SBPase (Fig. 5a). This coincides with the accumulation of FBP and depletion of F6P in the same strains. These results suggest that the FBP/SBPase, a bifunctional enzyme, may be a new flux control point in the engineered strains SA2 and SA3. As expected, this trend was reversed in the FBP/SBPase overexpressing strain SA7 (Fig. 5a) with substantial accumulation of S7P and depletion of SBP, suggesting that this is no longer a flux controlling step. However, now the bottleneck appears to have shifted to the enzyme RuBisCO as indicated by RuBP accumulation and 3-PGA depletion observed in SA7 (Fig. 5a).

In the strain SA7, metabolites such as glutamic acid (GLU), *N*-acetyl glutamate (N-Acetyl-GLU), γ-amino butyric acid (GABA) and proglutamate showed significant depletion compared to the wild-type (Fig. 5b). These metabolites are primarily derived from α-ketoglutarate (α-KG), which is the junction from where the flux for succinate is derived via the enzymes OgdA and SsaD suggesting a new metabolic bottleneck due to lower availability of α-KG.

Sucrose and its precursor sucrose-6-phosphate (S6P) show depletion while S6P’s precursor UDP-glucose shows accumulation in the engineered strains SA2 and SA3 suggesting that the sucrose phosphate synthase may be down-regulated in response to the metabolic alterations (Fig. 5c). Likewise, ADP-glucose and several other sugar nucleotides show accumulation in the engineered strains (Fig. 5c, d). These are considered to be the charged precursors for the synthesis of carbohydrate storage molecules or components of cellular structure and their accumulation may be indicative of the down-regulation of the steps that commit the carbon toward specific terminal metabolites. Note that the accumulation of ADP-glucose is more pronounced in SA7 as is expected because of the *glgA* knockout in this strain.

Interestingly, several of the nucleotide monophosphates such as AMP, IMP, GMP, CMP, UMP and deoxy-TMP show substantial depletion in the strain SA7 (Fig. 5e). Note that majority of the nucleotide di- and tri-phosphates do not show statistically significant change in the engineered strains. Further, the energy charge calculated based on ATP/(ATP + ADP) does not change significantly between the different strains. Thus, the change in the nucleotide monophosphates needs further investigation. Finally, significant changes were observed in molecules such as oxidized glutathione, NAD+ and NADP+ (Fig. 5f). Note that our method of extraction is expected to convert the reduced glutathione to oxidized glutathione [52]. Thus, the total glutathione pool in the recombinant strains is 2- to 3-fold higher than in WT. Moreover, the pool size changes in NAD+ and NADP+ may require further studies to correlate with other physiological characteristics.

**Fig. 6:**
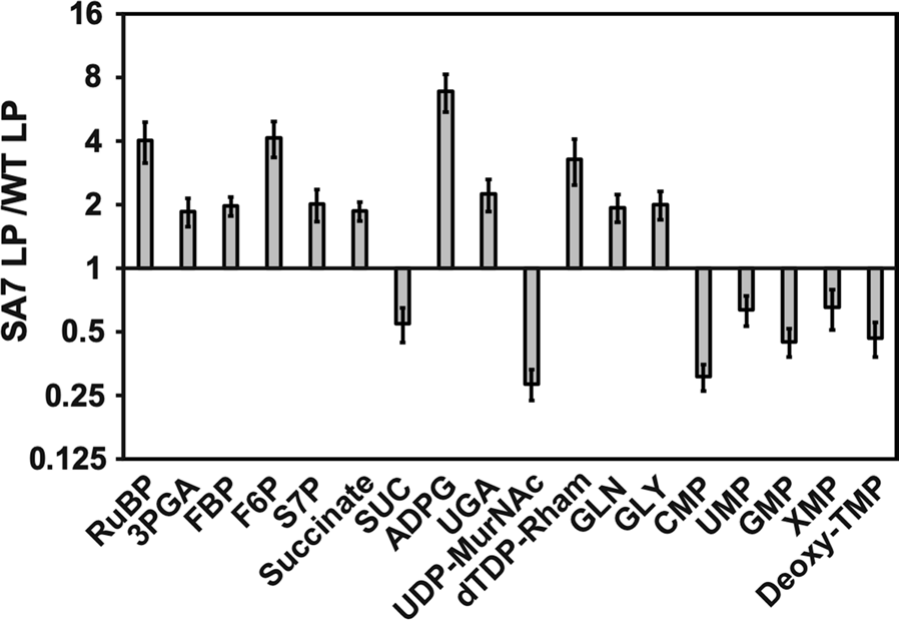
Metabolome profiling of *S. elongatus* SA7 in stationary growth phase. Cyanobacterial strains, SA7 and WT, were grown at 1% CO_2_ and metabolite extraction was carried out at stationary phase on day 3 (OD_730_ ~ 10). Only the metabolites that show significant difference between SA7 and WT are shown. Other details as shown in legend to Fig. 5

Since the rate of production decreased significantly beyond the third day of culture, we analyzed the metabolome of PCC 11801 SA7 with respect to wild-type on day 3 of the culture (Fig. 6). Of the 59 metabolites analyzed, 16 metabolites show a statistically significant difference between SA7 and WT (Fig. 6). Broadly, the trends observed during the exponential phase are also observed here. For example, the accumulation of S7P and relatively higher accumulation of F6P than FBP suggests that the bifunctional SBPase/FBPase enzyme is more active in SA7 than in WT. Further, RUBP shows greater accumulation than 3-PGA suggesting that RuBisCO is still a potential flux controlling step in SA7 at late stage. Some of the sugar nucleotides show accumulation while many of the nucleotide monophosphates show depletion as was observed during the exponential growth phase. Thus, the metabolome profile on day 3 has not provided any information as to why the succinate productivity declines beyond the third day.

#### Morphological analysis

Metabolome profile has revealed downshift of N-Ac-muraminate and N-acetyl glucosamine-6-P in the strain SA7, which are involved in the peptidoglycan biosynthesis. Further, significant changes were observed in several of the nucleotide sugars, which are precursors of storage molecules or structural components. Therefore, it was of interest to examine if the recombinant strains show any morphological alterations. During the initial phase of growth, the strain SA7 shows cell size similar to WT (Additional file 1: Figure S14). At subsequent stages of the growth, SA7 shows two to threefold elongation compared to the wild-type cells. It has been reported that cyanobacteria and other Gram-negative bacteria postpone cell division thereby forming long filaments under stress conditions [53]. We believe that intercellular metabolic imbalance in SA7 results in the reduction of septation frequency, thereby increasing the cell size. Nonetheless, the altered morphology of SA7 is expected to be advantageous for industrial applications with possible ease of biomass recovery [54].

## Discussion

We engineered the fast-growing and high light and temperature-tolerant cyanobacterium *S. elongatus* PCC 11801 to produce succinic acid. Building upon the previous reports on metabolic engineering of the model cyanobacterium *S. elongatus* PCC 7942 [28], we obtained a 2.2-fold improvement in succinate titer and ~ 40% reduction in the cultivation time over the best reported values for photoautotrophic growth [28] (Fig. 2). This was achieved by identifying the potential flux controlling steps based on metabolome profiling, enzyme assays and qualitative analysis of the transcript levels. Further, between the engineered strains PCC 11801 SA3 and PCC 7942 SA3 that carry identical genetic constructs integrated at the corresponding NSI sites, the former showed 40% higher succinate titer. This suggests that the fast growth rate of PCC 11801 can be potentially converted to higher product formation rates and that this new strain should be explored for other metabolic engineering studies. Further, the succinate titer was higher when grown under 1% CO_2_, a growth condition that results in metabolically more active culture as has been shown by up-regulation of several key enzymes [55]. We used the oxidative branch of the TCA cycle to produce succinate with an engineering strategy aimed at improving flux through this cycle that is normally not highly active during photoautotrophic growth [48]. Recently, dark fermentation of photoautotrophic grown and concentrated biomass of the cyanobacterium *Synechocystis* sp. PCC 6803 has been shown as a potential succinate production strategy [30, 31]. While the succinate titer in the fermentation medium was ~ 1.8 g l^−1^, the titer normalized on the photoautotrophic growth volume was much lower.

The concepts of metabolic flux control analysis [56–58] were qualitatively applied to identify the potential flux control points as targets for metabolic engineering. We performed extensive, targeted profiling of 59 metabolites to estimate the changes in pool sizes in the engineered strains with respect to the WT (Fig. 5). Metabolite abundances varied by up to 16-fold between the engineered and WT strains and provided vital clues about the potential rate-limiting steps. For example, accumulation of the reactant and depletion of the product of a reaction with respect to the wild-type suggests that the step may be flux controlling in the engineered strain. Our results show that new flux controlling steps emerged after overexpressing the enzymes of the previously identified bottlenecks. To exemplify, SBPase was identified as a flux controlling step in SA3. An attempt to address this limitation by overexpression of this enzyme in SA7 leads to the emergence of RuBisCO as the new rate-limiting step. We believe that this exercise of debottlenecking needs to be repeated to eliminate the potential rate-limiting steps and obtain a superior strain.

Photoautotrophic production with engineered cyanobacteria typically yields lower specific productivities than the heterotrophic organisms such as *E. coli* and yeast. We believe that there are several avenues for improvement of photoautotrophic succinate titer. Above certain cell density, cyanobacteria cultures are limited by light thereby leading to a decline in the productivity [59]. Therefore, it would be of interest to regulate the carbon partitioning between the biomass and the product of interest. An ideal photoautotrophic cell factory should grow to an optimal cell density and then continue to make the product without further cell growth [49]. In recent reports, targeted repression of genes *gltA,* pyruvate dehydrogenase (*PDH*) or Orotidine 5′-phosphate decarboxylase (*pyrF*) was performed to arrest growth at a predetermined cell density thereby improving carbon partitioning in favor of the desired product. In the present study, the carbon moles in succinate as a fraction of the total fixed carbon declined after the first 48 h of culturing thus resulting in less than 25% of the fixed carbon as product on the 5th day. This appears to be a common theme with engineered cyanobacteria [60]. The fast-growing *S. elongatus* PCC 11801 has a greater carbon fixation capacity, which needs to be harnessed for better product formation. Targeted repression of *gltA* cannot be performed since higher flux through the TCA is required for succinate production. However, the genes such as *pyrF* can be modulated for enhancing succinate titers by growth repression. The present work shows significant improvement over the best reported photoautotrophic succinate titer. In this study, a limited number of enzymes and genes were analyzed via enzyme assays and RT-PCR, respectively. Thus, genome wide transcriptomic and proteomic studies might provide additional clues on the consequences of pathway engineering. These, in turn, may be used to remove additional bottlenecks. Further, our LC/MS method was optimized for polar and charged intermediate metabolites. Some of the organic acids and amino acids were not detected in the present method. Thus, more extensive metabolomics studies may provide additional insights into the potential flux controlling steps not identified in this study. While the native promoters of PCC 7942 were used, it may be important to assess the effect of native promoters of PCC 11801 or inducible and tunable promoters. Also, extensive screening of promoters may be needed as it may not be feasible to predict the optimal promoter strength needed. Finally, the strain does not show any growth defect up to 125 mM succinate and hence at the current level of succinate titer, the product toxicity is not a concern. However, it would be useful to design strains that may tolerate even higher levels of succinate.

## Conclusion

We have engineered the novel cyanobacterium *Synechococcus elongatus* PCC 11801 for the production of succinic acid. We achieved a photoautotrophic succinic acid titer of 0.93 g l^−1^, which was 2.2-fold higher than the best reported titer. The studies of metabolomics, enzyme activity and transcript levels revealed the potential flux control points of the pathway. The bottlenecks were fixed by knocking out *glgA* and *sdhB* and by overexpressing *SBPase*, *gltA* and succinate transporters. However, several other bottlenecks were observed such as the RuBisCO enzyme. The use of native promoters over the inducible promoters is an added advantage as this will avoid the use of expensive inducers such as IPTG and in turn may improve the economic viability of the process under outdoor conditions. While this work shows significant improvement over the best reported photoautotrophic succinate titer, it is still a long way to go before the process can become commercially viable. Significant improvements in titer and productivity will be required. While the fast-growing *S. elongatus* PCC 11801 has a greater carbon fixation capacity, this needs to be harnessed for better product formation.

## Materials and methods

### Materials

All the chemicals used in this work were purchased from Sigma-Aldrich (St. Louis, MO) and Merck (Burlington, MA). The molecular biology kits were obtained from Qiagen (Hilden, Germany). Q5 DNA polymerase was purchased from New England Biolabs (Ipswich, MA).

### Methods

#### Culture and growth conditions

All the *S. elongatus* strains were grown in BG-11 medium (initial pH 7.5) in a shaker (Adolf Kuhner AG, LT-X, Birsfelden, Switzerland) at 38 °C, 120 rpm, and constant light intensity of 300 µmole photons m^−2^ s^−1^, unless specified otherwise. The cultivations under ambient air were with or without 50 mM NaHCO_3_ while those grown under 1% CO_2_ in the chamber were without added NaHCO_3_. Cell growth was monitored as optical density at 730 nm (OD_730_) using a spectrophotometer (Shimadzu, UV-2600, Singapore). To study the effect of light intensity and diurnal light, cultivations were carried out in a Multi-cultivator (Photon Systems Instruments, MC 1000-OD, Czech Republic) with 1% CO_2_ bubbling at a flow rate of 100 SCCM for each tube and OD_720_ recorded online.

#### Plasmid and strain construction

Summary of the nine recombinant strains of PCC 11801, SA1-SA9, is provided in Table 1 with additional details of the promoters, terminators, antibiotic cassettes in Additional file 1: Table S1, list of plasmids in Additional file 1: Table S2, plasmid maps in Additional file 1: Figure S3 and the primer sequences in Additional file 1: Table S4. The widely reported PCC 7942 plasmids pSYN1, pSYN6, pAM1619 [61] and pAM3558 [62] were procured from Life Technologies, California, USA and Addgene, Massachusetts, USA and used as the starting points. The plasmids pSYN1_A, pSYN6_B and pSYN6_C (in short, plasmids A, B and C) have the homology arms of NSI PCC 7942, which were used to transform both PCC 7942 and PCC 11801. Note that the parent plasmid pSYN6 has the PsbA1 promoter and a 6x-Histidine tag upstream of the *OgdA* gene. The remaining plasmids of Additional file 1: Table S2 carry the homology arms of PCC 11801 and were used to transform PCC 11801 alone. After genetic manipulations, the plasmids were first propagated in *E. coli* XL10 and isolated for transformation in cyanobacteria. The genes *SsaD*, *yjjP* and *yjjB* were amplified from *E. coli* MG1655. The gene *OgdA* of *Synechococcus* PCC 7002 was custom synthesized with codon optimization and procured from Invitrogen (Carlsbad, CA). The genes *gltA, SBPase* and *PEPC* and the native promoters and terminators used in this study were amplified from the genomic DNA of *S. elongatus* PCC 7942. The plasmids A and B were constructed by overlap extension PCR of the respective fragments, performed in two steps, and ligated into the *HindIII* and *Xho1* sites of the vectors pSYN1 and pSYN6, respectively. For plasmid C, *PEPC* along with promoter and terminator were joined using Circular polymerase extension cloning (CEPEC) and ligated into *Xho1* and *EcoRV* sites of plasmid B. For construction of *glgA* knockout plasmid, the homology arms of neutral sites II of pAM1619 were first replaced by the upstream and downstream regions of *glgA*, ~ 750 bp each, amplified from the genomic DNA of PCC 11801 to yield plasmid pAM1619_D. Additionally, the genes *LuxCDE* were removed from the plasmid pAM1619 using restriction digestion. Subsequently, the plasmid D was modified by ligating a fragment obtained by joining the promoter, *gltA* and terminator in the restriction sites *EcoRI* and *KpnI* to obtain pAM1619_E. Likewise, a promoter, *SBPase* and terminator were ligated into the restriction sites *KpnI* and *NheI* of plasmid_D followed by ligation of *gltA* construct to obtain pAM1619_F. The plasmid pAM3558_G was constructed by replacing the NSI homology arms of PCC 7942 with the upstream and downstream regions of *sdhB*, 500 bp each, using Polymerase Incomplete Primer Extension (PIPE) method as described [63]. Further, *E. coli* succinate transporter genes *yjjP* and *yjjB* under the control of P_rbc300_^Mutant^ (unpublished data, Additional file 1: Table S1) and P_cpcB200_ were joined and integrated into the pAM3558 to obtain pAM3558_H (Additional file 1: Figure S3). Plasmids were confirmed by PCR amplification of the individual genes and complete inserts, restriction digestion and sequencing.

#### Transformation

The recombinant strains of PCC 11801 SA1–SA9 were constructed by transforming the parental strains with the respective plasmid in the order shown in Additional file 1: Table S4. This requires that chromosomal segregation be achieved for the parental strain before initiating the next transformation. PCC 11801 is known to take up DNA naturally [24] and hence natural transformation was attempted first for each of the plasmids and was successful for plasmids A–F. For transformation with plasmids G and H, triparental conjugation was used as described [64] with modifications. For natural transformation, 7 ml of PCC 11801 or 2 ml of PCC 7942 culture of OD_730_ of 0.6 was centrifuged, washed and re-suspended in 100 µl BG-11 medium. To this, 500 ng of the desired plasmid was added and mixture incubated overnight under dark at 38  °C (for PCC 11801) and 34  °C (for PCC 7942). The mixture was then transferred to 20 ml BG-11 media containing 10 µg ml^−1^ spectinomycin (or 5 µg ml^−1^ kanamycin). The culture was incubated at 38  °C for 3–4 days and passaged in liquid several times by gradually increasing the antibiotic concentration to achieve complete chromosomal segregation. Then, the culture was spread on BG-11 Agar plates with 100 µg ml^−1^ spectinomycin or 50 µg ml^−1^ Kanamycin to obtain single colonies. Complete segregation was checked using confirmation primers (Additional file 1: Figure S2, Table S4).

#### Conjugation method of transformation

Conjugation was carried out using *E. coli* HB101 having pRL443 plasmid (Addgene) as described previously [65]. Twenty-five ml of SA7 or 6 ml of SA3 cultures of OD_730_ of ~ 0.6 were centrifuged and washed twice with 1 ml of BG-11 medium. Likewise, 2 ml overnight grown cultures each of *E. coli* HB101 pRL443 and *E. coli* TOP10 containing the plasmid of interest (i.e., pAM3558_G and pAM3558_H) were centrifuged and washed twice with 100 µl milliQ water. The three pellets were then resuspended and mixed gently in 200 µl of BG-11 medium and the mixture was spread on a membrane (47 mm, 0.4 µm Nuclepore Track-Etch, Whatman, Maidstone, UK) placed on a BG-11 agar plate supplemented with 5% Luria Broth and incubated for 24 h at 38 °C and 100 µE light until mat cyanobacterial growth appears on the filter paper. Filter papers were then transferred to BG-11 plates having 10 µg ml^−1^ of gentamicin and grown for 3 days at 38 °C and 100 µE light. Colony PCR was performed to select positive colonies, which were patched on higher antibiotic BG-11 plates to achieve complete chromosomal segregation.

#### Estimation of succinate titer

One ml of the cyanobacterial culture was drawn every 24 h up to 5 days, centrifuged and supernatant stored at − 20 °C until analysis. For routine measurements, the Succinic acid assay kit of Megazyme was used according to the manufacturers’ protocol. Briefly, 10 µl samples, diluted as required, were added to duplicate wells of a 96 well plate and other assay reagents added sequentially. Difference in absorbance at 340 nm was recorded to compute the succinate titer. A standard graph was created initially in the range of 50 to 400 mg l^−1^ and then one-point standard of 200 mg l^−1^ was used for routine assays. Additionally, the succinate titers for the strains PCC 11801 SA1-SA3 were validated by an independent method by using Succinate Colorimetric Assay Kit (Sigma-Aldrich, MO, USA) and by using the manufacturers’ protocol. Briefly, 1–50 µl samples were added into duplicate wells of a 96 well plate. Assay buffer was used to make up the volume to 50 µl. Additional 50 µl of the reaction mixture containing succinate enzyme mix, succinate substrate convertor and succinate developer was added and incubated at 37 °C for 30 min. The color developed was measured at 450 nm. The succinate titers with the two Assay kits matched within an error of 15%.

Further, for the representative strain PCC 11801 SA3, succinate product was confirmed and quantified by gas chromatography coupled to mass spectrometry (GC–MS). Briefly, the culture supernatant was collected, dried and derivatized as reported [17]. Succinate standards were prepared similarly. Analysis was carried out using GCMS-TQ8040 Triple Quadrupole instrument (Shimadzu, Kyoto, Japan) with DB-5 column of 0.25 µm film thickness × 0.25 mm diameter × 15 m length (Agilent Technologies, Santa Clara, USA). Helium was used as the carrier gas at a flow rate of 1.5 ml min^−1^. The oven temperature program was: 100 °C for 1 min, increased to 210 °C at a rate of 5 °C min^−1^, then increased to 300 °C at a rate of 30 °C min^−1^ and held for 6 min. Injection volume was 1 µl with a 10:1 split ratio. Other parameters: the injector temperature of 250 °C, the temperature of auxiliary transfer line of 300 °C. The concentration of succinate in the sample was calculated from the standard curve.

#### Enzyme assays

Enzyme assay protocols were adopted from those reported in literature and modified as necessary. All assays were performed in duplicate on two independent biological samples. For all the enzymatic assays, 50 ml of 0.6 OD_730_ culture grown under 1% CO_2_ was harvested and centrifuged. The pellet was re-suspended in 1 ml lysis buffer (100 mM Tris HCl, pH 7.4, containing 1 mM PMSF, 1 mg ml^−1^ lysozyme) and lysed using TissueLyser (Qiagen, Hilden, Germany) using 0.5-micron glass beads with 30 cycles of 1 min on, 1 min off-incubated on ice. The lysate was centrifuged at 13,000 rpm, 25 min, 4 °C and the clear supernatant were used for enzyme assays. Protein concentration was estimated with Bradford’s reagent (Bio-Rad Laboratories, CA, USA) and the activity was estimated as nanomoles/min/mg crude protein after adjusting for the substrate blank as the negative control.

The SsaD enzyme assay was a coupled assay using purified OgdA enzyme based on the reaction shown below and as reported [28] with minor modifications.

$$\propto {\text{Ketoglutarate}}\mathop \to \limits^{\text{OgdA}} {\text{Succinate}}\;\;{\text{Semialdehyde}} + {\text{CO}}_{2}$$

$${\text{Succinate}}\;\;{\text{Semialdehyde}} + {\text{NADP}} \mathop \to \limits^{\text{SsaD}} {\text{Succinate}} + {\text{NADPH}}$$

Briefly, the reaction mixture comprising 100 mM Tris HCl, pH 7.4, 1 mM MgCl_2_, 10 mM α-ketoglutarate, 0.2 mM NADP+, 0.3 mM Thiamine Pyrophosphate and purified OgdA enzyme were incubated for an hour at room temperature. Then, 1 mM NADP+ and 10–50 μl of cyanobacterial lysate was added to the mixture. The accumulation of NAPDH was monitored at 340 nm.

Initial trials for the expression of OgdA in *E. coli* BL21 (DE3) resulted in an inactive protein in inclusion bodies (data not shown). Therefore, as reported [66], OgdA was co-expressed in *E. coli* BL21 (DE3) with the chaperone trigger factor, Ptf16 (Takara Bio Inc., Japan) to obtain expression in the soluble fraction. The competent cells were initially transformed with plasmid Ptf16, followed by transformation with pET21a containing the *OgdA* gene. A positive clone was selected and cultivated in liquid LB media containing carbenicillin (100 μg ml^−1^) and chloramphenicol (25 μg ml^−1^) at 37 °C, 180 rpm. One percent of the overnight grown culture was inoculated in LB medium and l-(+)-arabinose (1 mg ml^−1^) was added to induce the Ptf16. After reaching OD_600_ ~ 0.6, OgdA expression was induced with 0.2 mM isopropyl-β-d-thiogalactopyranoside (IPTG) at 16 °C, overnight. The culture was centrifuged, re-suspended in ice-cold lysis buffer (50 mM NaH_2_PO_4_, 300 mM NaCl, pH 7.4, 1 mg ml^−1^ lysozyme, 1 mM PMSF) and sonicated (QSonica, Newtown, USA). The lysate was centrifuged at 15,000 rpm and 4 °C for 1 h and the supernatant loaded onto a Ni–NTA column (Qiagen Hilden, Germany) pre-equilibrated with 50 mM NaH_2_PO_4_, 300 mM NaCl, pH 7.4. The enzyme was eluted with 250 mM Imidazole and fractions with the highest specific activity pooled.

PEP carboxylase assay was conducted as a coupled assay with purified malate dehydrogenase (mdh) from *E. coli* as reported [28].

$${\text{Phosphoenol}}\;\;{\text{pyruvate}} + {\text{CO}}_{2} \mathop \to \limits^{\text{PEPC}} {\text{Oxaloacetate}} + {\text{ADP}}$$

$${\text{Oxaloacetate}} + {\text{NADH}} \mathop \to \limits^{\text{mdh}} {\text{Malata}} + {\text{NAD}}$$

The reaction mixture comprised 10 mM NaHCO_3_, 400 μM NADH, 5 μg of purified mdh, 5 mM PEP, and 10-50 μl cyanobacterial lysate in 100 mM Tris–HCl buffer pH 7.5. The rate of decrease of absorbance at 340 nm.

For purification of malate dehydrogenase (mdh), *mdh* was amplified from *E. coli* MG1655 strain and cloned into pET21a. The plasmid was transformed in *E. coli* BL21 (DE3) and expressed by induction with 1 mM IPTG, at 25 °C, overnight. The culture was centrifuged and re-suspended in ice-cold lysis buffer and sonicated. The cell lysate was centrifuged and the supernatant loaded into a Q-Sepharose (GE health care) pre-equilibrated with 50 mM NaH_2_PO_4_, 50 mM NaCl, pH 7.4. Fractions were eluted in the buffer with 50-400 mM NaCl and those with high specific activity were pooled together and concentrated in 20 mM potassium phosphate saline, pH 7.4. The mdh activity was assayed and calculated by monitoring the reduction of NADPH. The reaction mixture contained 100 mM Tris HCl, pH 7.2, 0.15 mM NADH, 0.2 mM oxaloacetate along with various dilutions of the purified mdh.

Citrate synthase assay was conducted as described [67] with minor modifications. Briefly, this assay monitors the release of free CoA by reacting it with 5,5′-dithiobis-(2-nitrobenzoic acid) (DTNB), which reacts with free thiols to generate a yellow color. The reaction was carried out in 100 mM Tris–HCl buffer pH 7.5 containing 200 μM acetyl-CoA, 1 mM oxaloacetate, 100 μM DTNB, and cyanobacterial lysate. The liberation of CoA was monitored spectrophotometrically at 412 nm. Standard curve was constructed using free CoA with 100 μM DTNB.

Isocitrate dehydrogenase assay was conducted as described [28] with minor modifications. The increase in absorbance at 340 nm due to the formation of NADPH, corresponds to the conversion of isocitrate to α-ketoglutarate. The reaction was carried out in 100 mM Tris HCl buffer pH 7.5 containing 3 mM MgSO4, 1 mM NADP ^+^ , 5 mM dl-isocitrate, and the cyanobacterial lysate.

### RNA isolation and reverse transcriptase-PCR

RNA was isolated using GenElute™ Total RNA Purification Kit (Sigma-Aldrich, MO, USA). For genomic DNA removal, TURBO DNA free kit (Thermo Fischer, USA) was used according to the manufacturer’s instructions. cDNA synthesis was performed using an iScript Advanced cDNA synthesis kit (Bio-Rad Laboratories, CA, USA) according to the manufacturer’s instructions using a mixture of oligo-dT and random hexamer primers. Subsequently, reverse transcriptase-PCR (RT-PCR) was carried out from the synthesized cDNA using primers listed in the Additional file 1: Table S4 that can amplify ~ 200 base pair amplicons. PCR was performed with 30 cycles: 30 s at 95 °C; annealing: 45 s at 60 °C; and extension 60 s at 72 °C. The PCR products were loaded on the 1.2% agarose gel and visualized under UV trans-illuminator.

### Metabolite pool size analysis using LC–MS

#### Preparation of samples for metabolomics analysis

Sampling and metabolite extraction was performed as described previously [68, 69]. Twenty ml of ~ 0.6 OD_730_ culture was filtered rapidly through nylon membrane filter (Whatman, 0.8 µ, catalog no. 7408-004) in the presence of light. Smaller volumes were used while collecting the late phase samples of higher OD_730_ to maintain match the dry cell weight in the different metabolomics experiments. The cells on the membrane filters were quickly transferred to 80:20 methanol–water v/v (precooled to − 80 °C) chloroform was added and vortexed for 25 min in a cycle of 1 min vortex and 1 min cooling on ice. Finally, 0.2 M ice-cold ammonium hydroxide was added and the samples were vortexed for 10 min. The samples were centrifuged at 8000*g* and the aqueous phase was lyophilized and stored at − 80 °C until ready for LCMS analysis.

#### LCMS sample preparation and data acquisition

The metabolite extract of each test sample was mixed with an equal volume of an extract of the PCC 11801 WT that was fully labeled with ^13^C isotopic carbon by growing for ~ 5 generations in the presence of NaH^13^CO_3_ in modified BG-11 medium. The fully ^13^C labeled metabolite extract served as internal standard. The modified BG-11 medium did not contain any organic carbon source such as sodium carbonate, citric acid, and ferric ammonium citrate. Iron sulfate heptahydrate was used as iron source. The metabolites were separated using reverse-phase ion pairing chromatography using C18 Synergi 4 μm Hydro-RP LC column 150 × 2 mm (Phenomenex Inc, Torrance, CA). The injection volume was 6 µl. The gradient elution method comprised eluents 10 mM tributylamine + 15 mM acetic acid in water (pH = 4.95) (buffer A) and 100% Methanol (buffer B). The gradient method used is as follows: 0% B (0.01 min), 0% B (2 min), 35% B (8 min), 35% B (10.5 min), 90% B (15.50 min), 90% B (20.5 min) and 0% B (22 min). The column temperature and flow rates were 25 °C and 0.3 ml min^−1^, respectively. The data was acquired using information dependent acquisition (IDA) method on Q-TOF instrument (TripleTOF 5600, SCIEX, Framingham, MA) interfaced with Shimadzu Ultra Performance-Liquid Chromatography (UPLC) system (Shimadzu, Nexera LC-30 AD, Singapore). The instrument was operated under negative ion mode. The curtain gas, gas 1 and gas 2 were kept at 35, 40 and 40 psi, respectively. The ion source temperature was 450 °C and the voltage was − 4500 V.

#### LCMS data analysis

The metabolites presented in this study were identified at MS2 level using MS-DIAL and MetDIA tools [70, 71]. Approximately 50% of these identified metabolites were also confirmed by injection of pure LCMS grade standards. MultiQuant 3.0.1 (SCIEX, Framingham, MA) was used to quantify the peak areas corresponding to the ^12^C and ^13^C monoisotopic peaks for the metabolites of interest. The former corresponds to the metabolite in the test sample while the former corresponds to that in the internal standard. The area ratios thus calculated provide normalized pool sizes and can be readily compared across the different samples.

#### Fluorescence microscopy

Cultures of OD_730_ 0.8, 5, 10 were centrifuged at 5000*g* for 3 min. The pellet was washed with milli-Q water twice and then re-suspended in 4% formaldehyde. The fixed cells were mounted on the slide and observed under a fluorescence microscope, Zeiss Axio Observer Z1 (100× objectives, NA = 1.40; Carl Zeiss MicroImaging Inc., Oberkochen, Germany) equipped with Axiocam camera controlled by Axiovision software [Axio Vision Release 4.8.3 SP1 (06–2012)]. Exposure time for imaging was 300 ms.

### Statistical analysis

Statistical significance of the measured parameters such as succinate and fumarate titers and enzyme levels among different engineered strains was examined by one-way ANOVA, followed by Tukey multiple-comparison test. *p*-value ≤ 0.05 was considered as statistically significant during the above comparison. For metabolomics study, student t-test was performed to compare the metabolite levels between the WT and the recombinant strains. Metabolites with a fold change of ≥ 1.5 or ≤ 0.66 between the engineered and to WT strains and *p*-value ≤ 0.05 were considered as statistically significant. Other statistical analysis such as principal component analysis and generation of heat maps and volcano plots were performed using MetaboAnalyst 4.0 [72, 73]. The standard error of the mean (SEM) of ratio of metabolite levels between the engineered and WT strains, denoted as *X* and *Y*, respectively, was calculated as follows. The variance of the ratio *X*/*Y* was first calculated using Taylor expansion method according to the following equation [74]:

$$\sigma^2{_{\frac{X}{Y}}} = \frac{1}{{\overline{{\bar{Y}^{2} }} }}\sigma_{X}^{2} + \frac{{\bar{X}^{2} }}{{\bar{Y}^{4} }} \sigma_{Y}^{2} - 2\frac{{\bar{X}}}{{\bar{Y}^{3} }} \text{cov} \left( {X,Y} \right),$$

where *σ*^2^ denotes the variance, *X* and *Y* denote two conditions, *X̅* and *Y̅* are the mean of the metabolite levels in the conditions *X* and *Y*, respectively, and cov (*X*, *Y*) denote the sample covariance. The fold change between two conditions is represented as

$$\frac{{\bar{X}}}{{\bar{Y}}} \pm \frac{{\sigma_{{\frac{X}{Y}}} }}{\sqrt n },$$

where n denotes the number of replicate measurements.

## Supplementary information

**Additional file 1.** Additional figures and tables.

## Data Availability

All data generated or analyzed during this study are included in this published article and its additional files. The LC/MS raw data files generated for the metabolomics study of engineered strains have been deposited with Metabolomics Workbench (http://www.metabolomicsworkbench.org/), doi: http://dx.doi.org/10.21228/M82M47. Area ratios of each metabolite and its respective ^13^C-labeled internal standard have been provided in the source data file for Figs. 5 and 6.

## Abbreviations

PEPC: PEP carboxylase
GltA: Citrate synthase
OgdA: 2-ketoglutarate decarboxylase
SsaD: NADP-dependent succinate semialdehyde dehydrogenase
sdhB: Succinate dehydrogenase subunit B
SBPase/FBPase: Fructose-1,6-biphosphatase/sedoheptulose-1,7-biphosphatase
glgA: Glycogen synthase
Glg: Glycogen
ADPG: Adenosine-5ʹ-diphosphoglucose
G1P: Glucose 1-phosphate
G6P: Glucose 6-phosphate
F6P: Fructose 6-phosphate
FBP: Fructose bisphosphatase
G3P: Glyceraldehyde 3-phosphate
3PGA: 3-Phosphoglyceric acid
PEP: Phosphoenolpyruvate
Pyr: Pyruvate
AcCoA: Acetyl-CoA
Cit: Citrate
ICI: Isocitrate
α-KG: Alpha ketoglutarate
SuccCoA: Succinyl CoA
Fum: Fumarate
Mal: Malate
OAA: Oxaloacetate
RuBP: Ribulose 1,5-bisphosphate
Ru5P: Ribose 5-phosphate
S7P: Sedoheptulose 7-phosphate
SBP: Sedoheptulose 1,7-bisphosphate
GOX: Glycolate oxidase
GLY: Glycine
GLU: Glutamate
6PG: 6-Phosphogluconate
Suc: Sucrose
Suc6P: Sucrose 6-phosphate
Glc6P: Glucose-6-phosphate
GlcPNAc: *N*-Acetylglucosamine
UDP-MurNAc: UDP-*N*-acetylmuramic acid
