# Supplementary material for: Metabolic engineering of a fast-growing cyanobacterium Synechococcus elongatus PCC 11801 for photoautotrophic production of succinic acid
Source: Biotechnol Biofuels. 2020 May 18;13:89. doi: 10.1186/s13068-020-01727-7 (PMC7236211; doi:10.1186/s13068-020-01727-7)
Supplement: Supplementary file 1 — Additional file 1. Additional figures and tables. [file 13068_2020_1727_MOESM1_ESM.docx]

**Additional Tables and Figures**

**Metabolic engineering of a fast-growing cyanobacterium *Synechococcus elongatus* PCC11801 for photoautotrophic production of succinic acid**

Shinjinee Sengupta^1^, Damini Jaiswal^1^, Annesha Sengupta^1^, Shikha Shah^1,2^, Shruti Gadagkar^1^ and Pramod P. Wangikar^123^

*^1^Department of Chemical Engineering, ^2^DBT-Pan IIT Center for Bioenergy, ^3^Wadhwani Research Center for Bioengineering, Indian Institute of Technology Bombay, India, Indian Institute of Technology Bombay, India*

*Corresponding author’s email address:* [wangikar@iitb.ac.in](mailto:wangikar@iitb.ac.in)


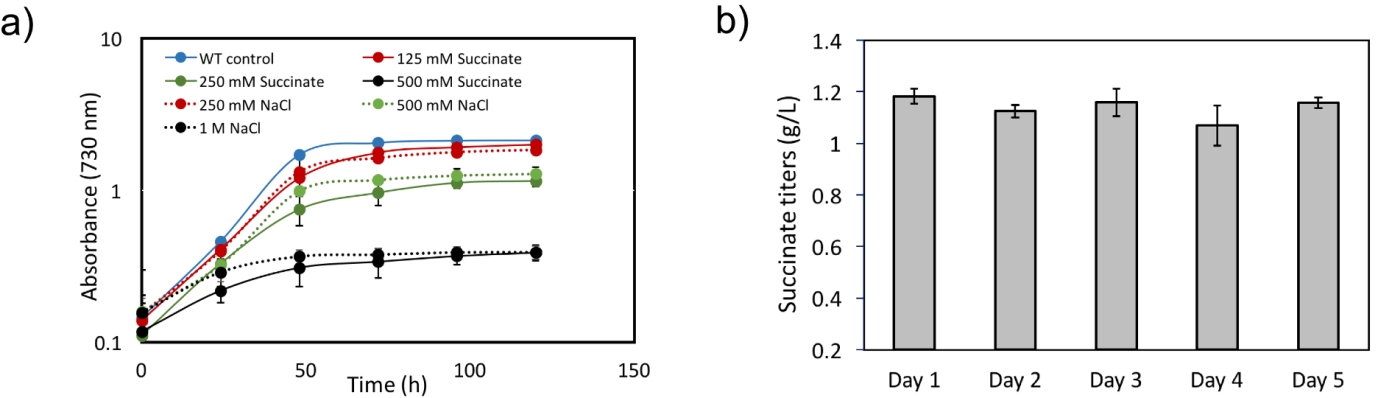


**Figure S1.** Effect of succinate on growth of *S. elongatus* PCC 11801. a) Growth was monitored in BG-11 medium containing different concentrations of disodium succinate and NaCl in shake flasks at 38°C and under ambient air. b) Concentration of succinate in culture medium upon incubation with *S. elongatus* PCC 11801.


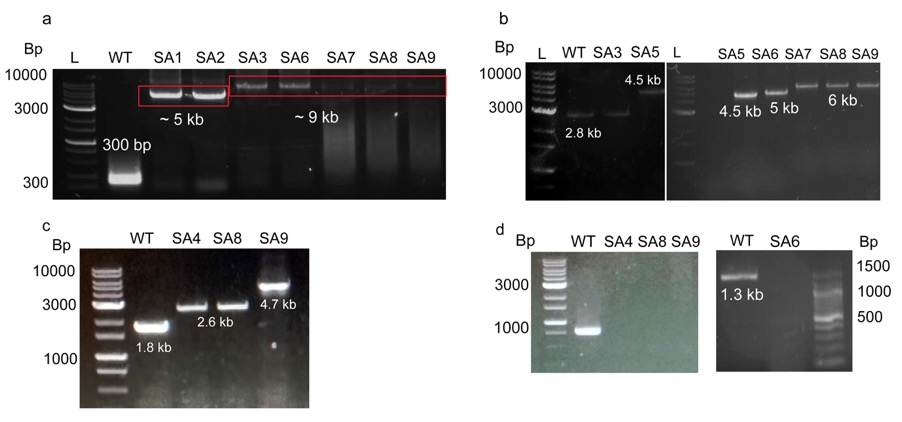


**Figure S2**. Verification of complete chromosomal segregation of the recombinant strains by using the primers for the respective target sites as shown in Supplementary Table 4. a) Chromosomal segregation of the recombinant strains at the neutral site I (NSI) checked by using the primers IITB 51 and IITB 52. Complete disappearance of the band at 300 bp and appearance of a band corresponding to the insert confirms complete chromosomal segregation. b) Chromosomal segregation of the recombinant strains at the *glgA* site checked by using the primers IITB 20 and IITB 21. c) Chromosomal segregation of the recombinant strains at the *sdhB* site checked by using primers IITB 47 and IITB 50. d) Confirmation of *sdhB* gene knockout by using primers IITB 55 and IITB 56 and *glgA* gene knockout by using primers IITB 53 and IITB 54 respectively. The chromosomal DNA was isolated by using the Megazyme DNA extraction kit.


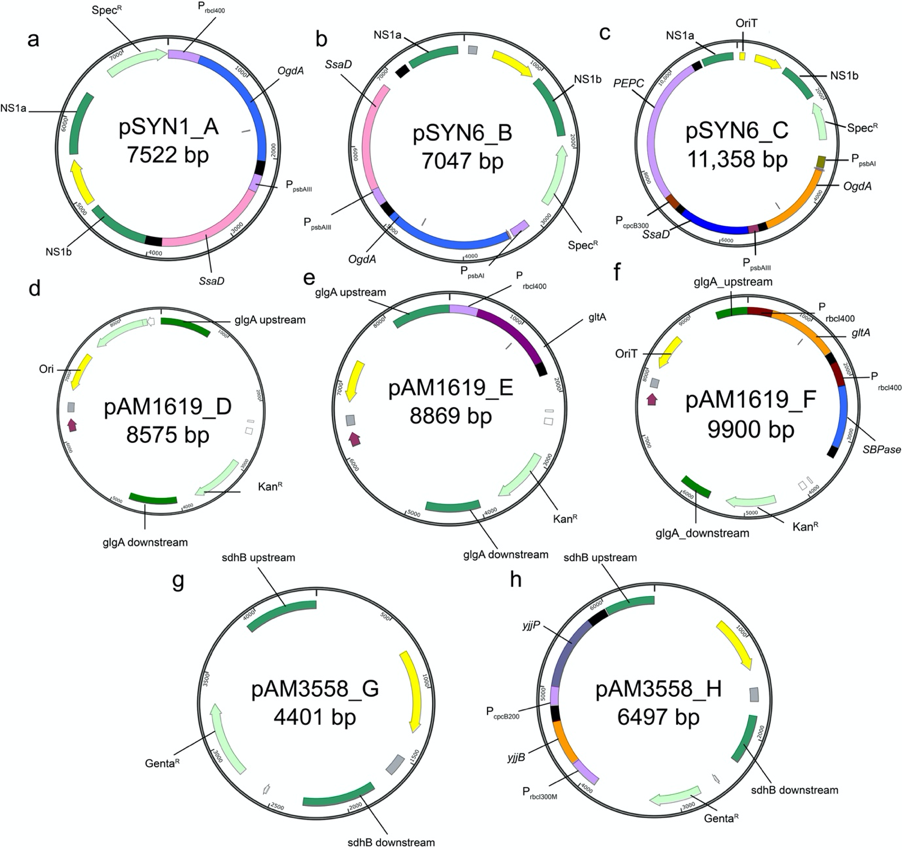


**Figure S3. a-h**- Maps of the eight plasmids constructed in this work. Details of the genetic constructs are provided in Supplementary Table 2. Briefly, each map shows the homology arms for the target sites (shown in dark green), the antibiotic markers (shown in light green), promoters (shown in light purple) and genes to be overexpressed. Terminators are shown as black bars.


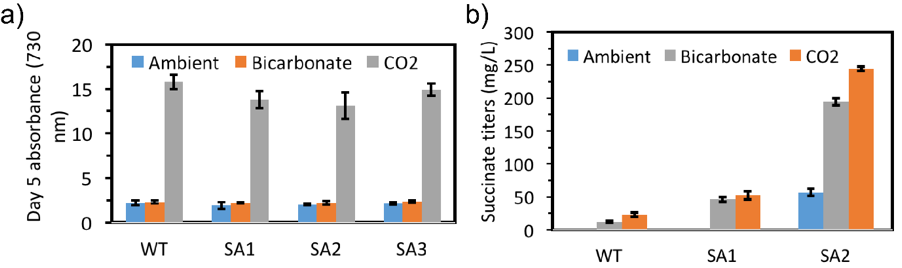


**Figure S4.** Biomass accumulation (a), and succinate production (b) on the 5^th^ day under ambient air without and with bicarbonate (50 mM NaHCO3) and under 1% CO_2_ without bicarbonate. Three representative strains SA1-SA3 were chosen for growth studies while the succinate titer was measured in SA1 and SA2 using the succinate assay kit (Sigma, St. Louis, MO).


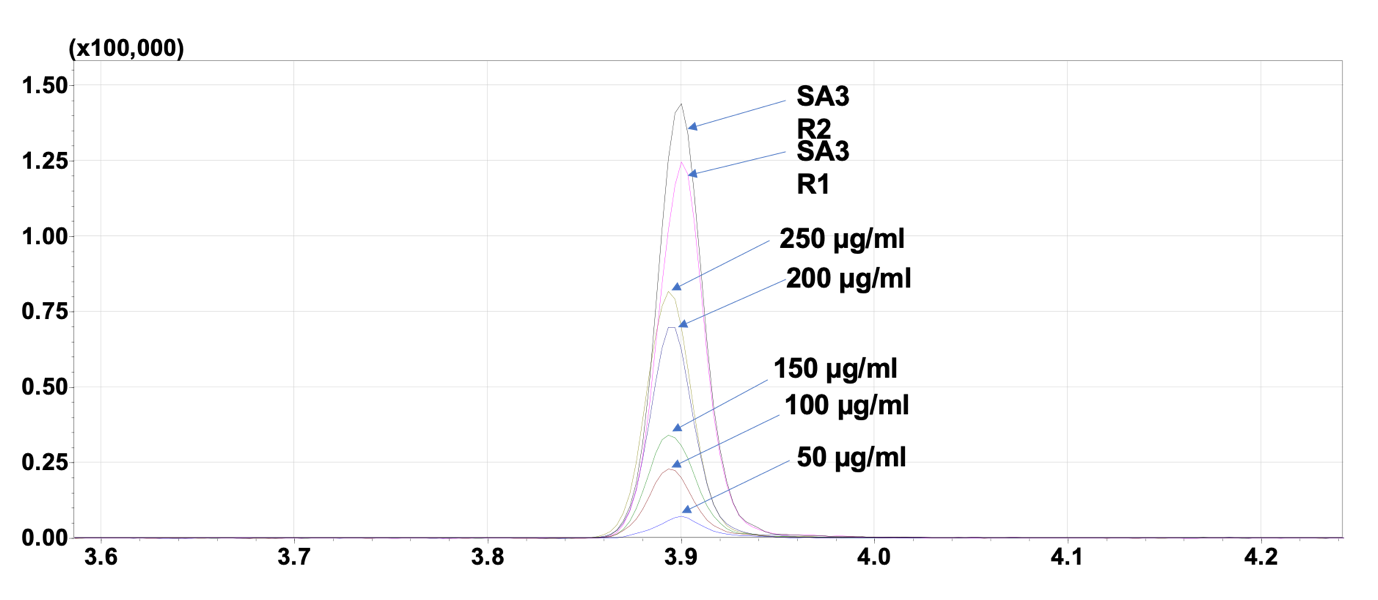


**Figure S5. End point product determination assay using GC-MS.** Succinate product formation for PCC 11801 SA3 was confirmed by gas chromatography coupled to mass spectrometry (GC-MS). The figure shows the extracted ion chromatogram (XIC) of different concentrations of succinate of standard and sample. Different concentrations of succinate standards from 50 μg/ml to 250 μg/ml were injected along with supernatant obtained from PCC 11801 SA3. R1 and R2 denotes two biological replicates. Arrow points to the respective peak in the analysis. Succinate was analyzed as trimethylsilyl derivatives after derivatization with MSTFA.


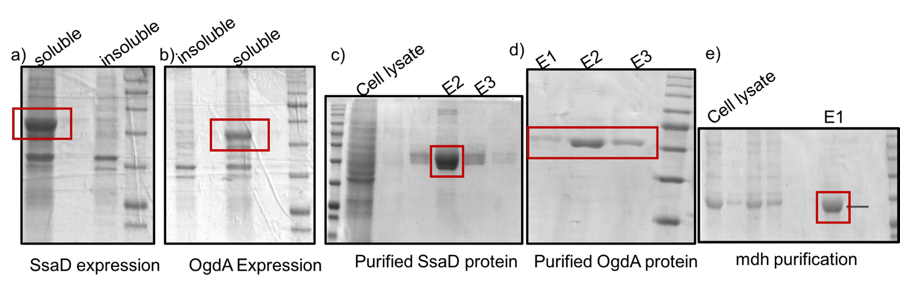


**Figure S6.** SDS-PAGE for expression and purification of enzymes SsaD, OgdA and mdh expressed in *E. coli* BL21. For the enzyme assays performed in the study, expression and purification of enzymes such as a) SsaD expression in the soluble fraction, b) OgdA expression in the solution fraction, c) SsaD purification, d) OgdA purification e) mdh purification. OgdA and SsaD was performed using Ni NTA affinity chromatography as mentioned in the material method section. mdh was purified using ion exchange chromatography as mentioned in the material method section. Purified OgdA and SsaD were used as positive control for SsaD coupled assay. E1- Elution 1, E2- Elution 2, E3- Elution 3.


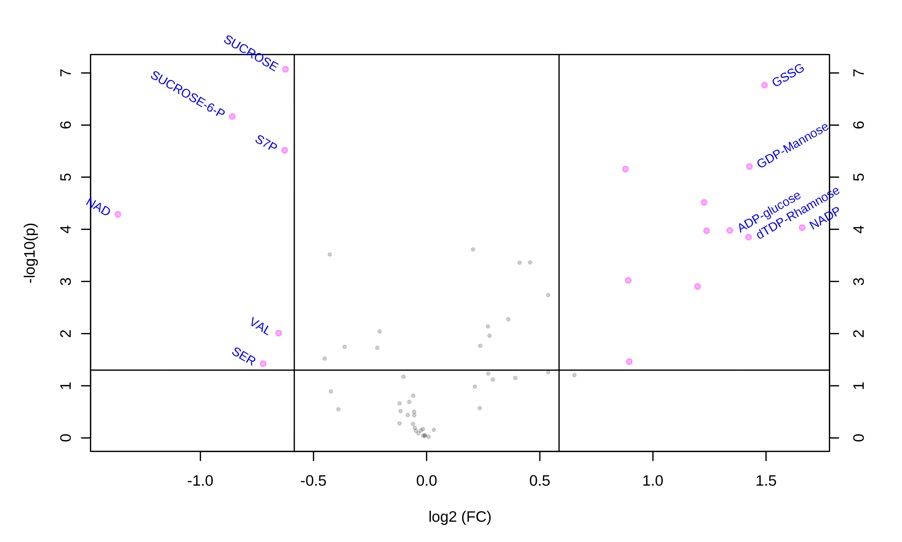


**Figure S7. Volcano plot for SA2**. The volcano plot with fold change (SA2/WT) threshold of 1.5 (x-axis) and t-tests threshold of 0.05 (y-axis). The pink circles represent the metabolites that are above the threshold. Both fold changes and p values are log transformed. The further its position away from the (0,0), the more significant the change is.

**
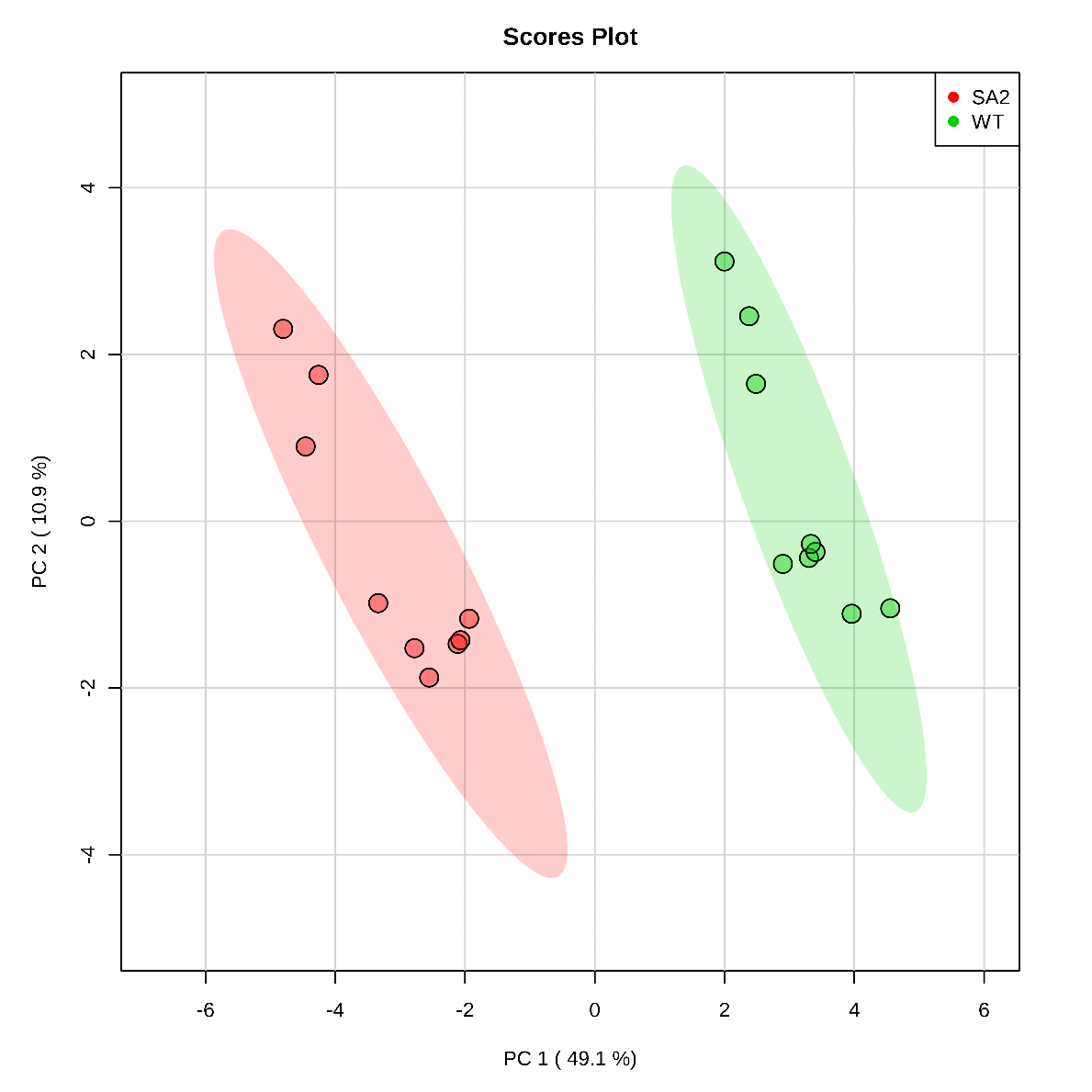
**

**Figure S8.** The principal component analysis (PCA) score plot with the metabolites data showing WT and SA2 as distinct groups. The area with 95% confidence intervals of each group is highlighted.


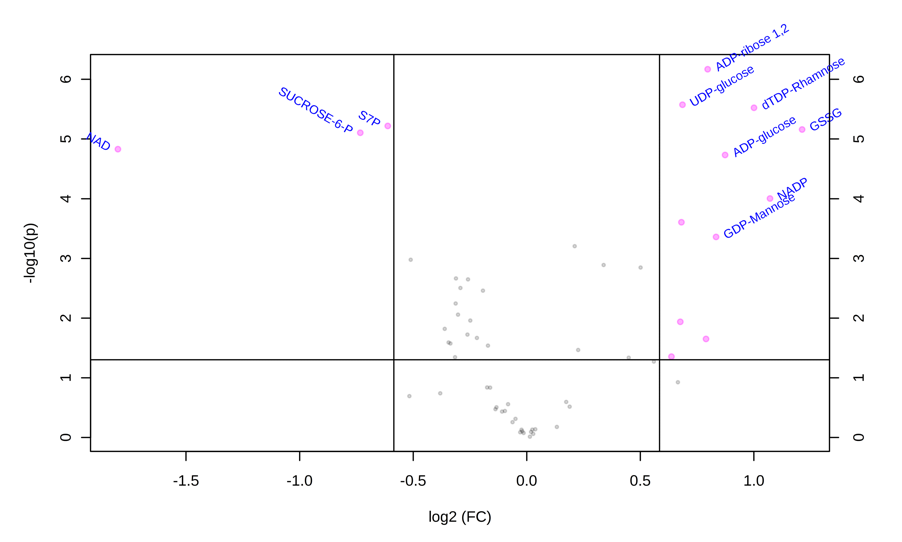


**Figure S9. Volcano plot for SA3**. The volcano plot with fold change (SA3/WT) threshold of 1.5 (x-axis) and t-tests threshold of 0.05 (y-axis). The pink circles represent the metabolites that are above the threshold. Both fold changes and p values are log transformed. The further its position away from the (0,0), the more significant the change is.


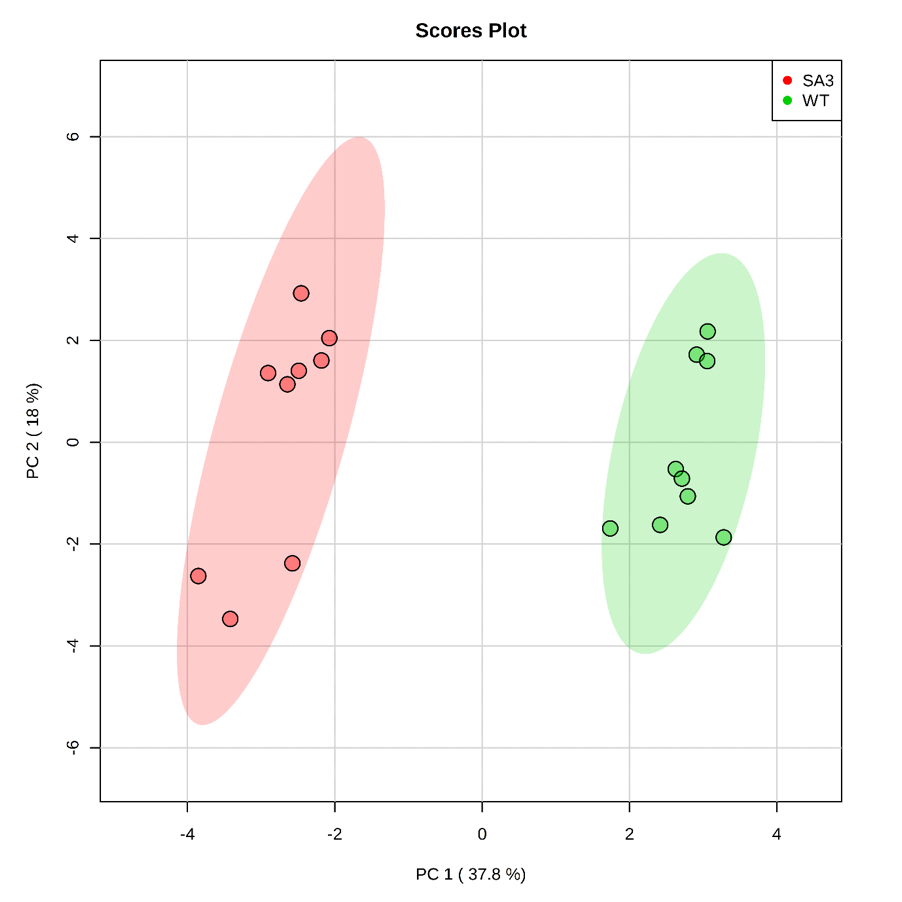


**Figure S10.** The principal component analysis (PCA) score plot with the metabolites data showing WT and SA3 as distinct groups. The area with 95% confidence intervals of each group is highlighted.


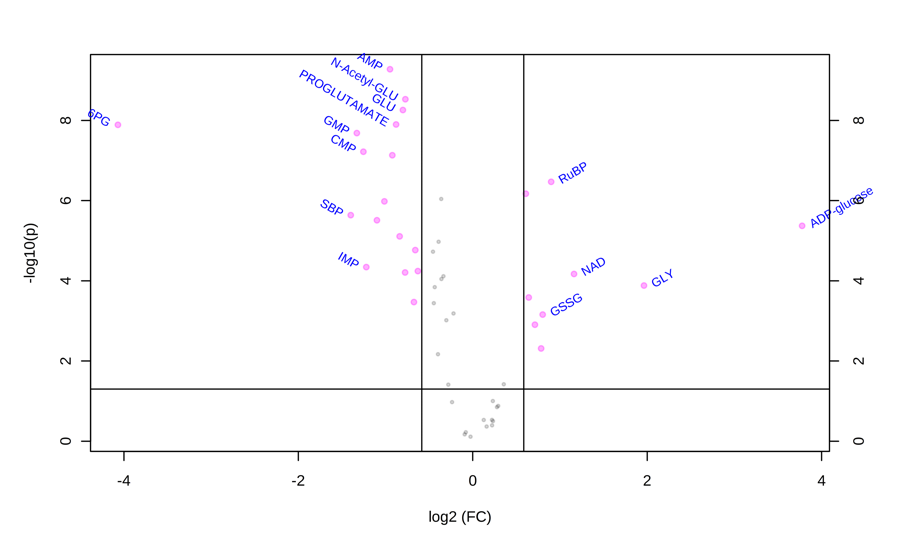


**Figure S11. Volcano plot for SA7.** The volcano plot with fold change (SA7/WT) threshold of 1.5 (x-axis) and t-tests threshold of 0.05 (y-axis). The pink circles represent the metabolites that are above the threshold. Both fold changes and p values are log transformed. The further its position away from the (0,0), the more significant the change is.


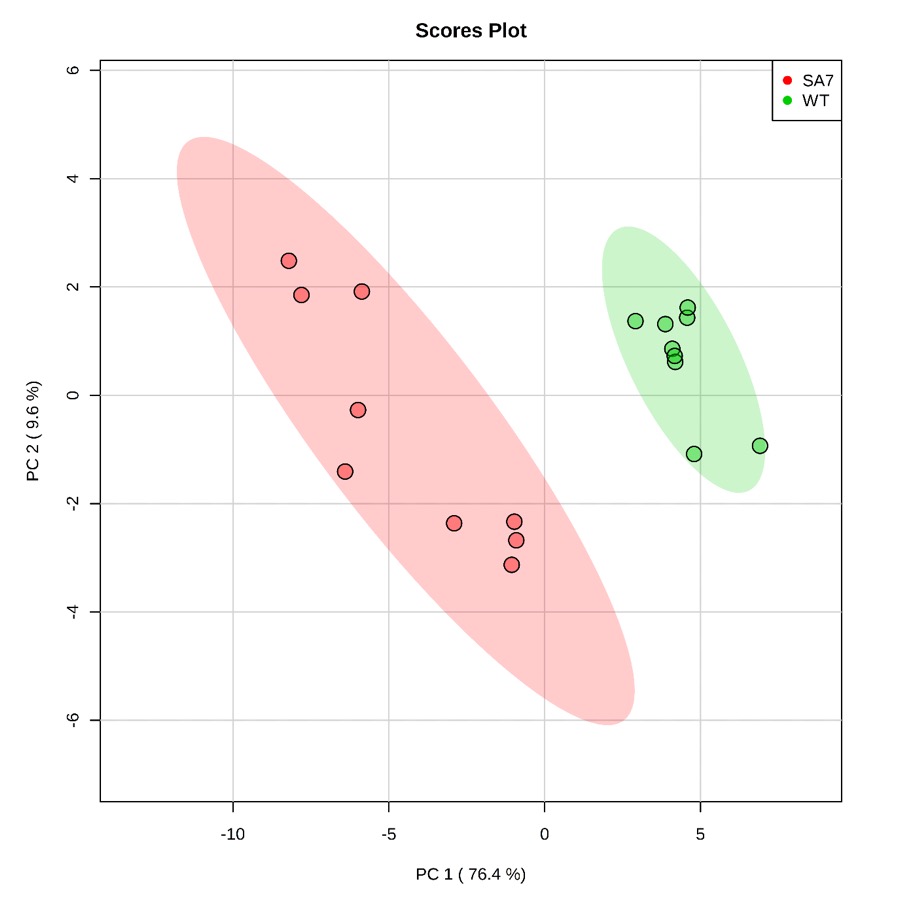


**Figure S12.** Score plot for SA7. The principal component analysis (PCA) score plot with the metabolites data showing WT and SA7 as distinct groups. The area with 95% confidence intervals of each group is highlighted.


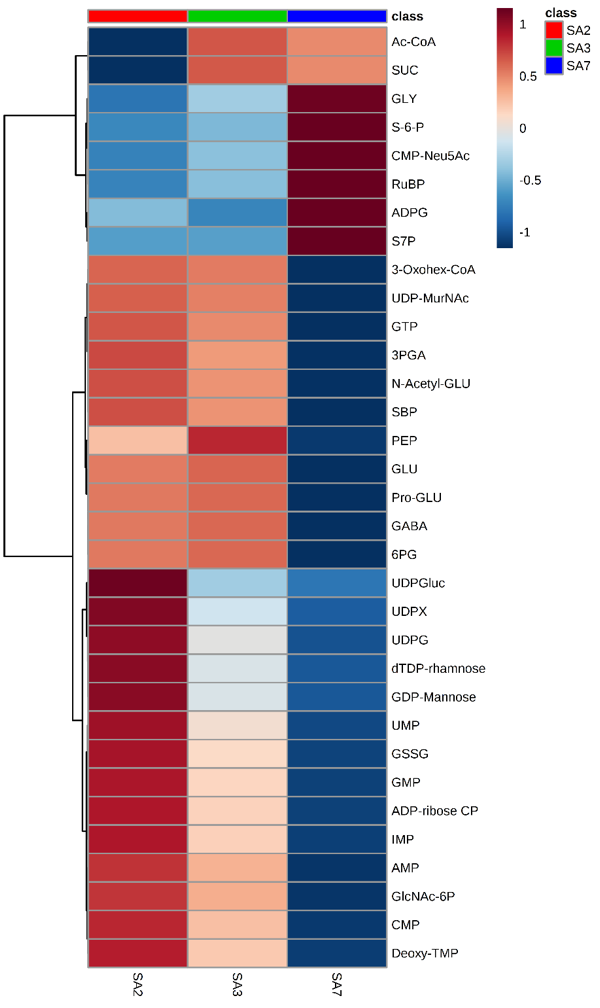


**Figure S13.** Heat map for all the metabolites showing significant fold change (fold change ≥1.5 or ≤0.66 and a p-value ≤0.05) in any of the three recombinant strains compared to the WT. The fold change values are log_2_ transformed and the heat map was created using MetaboAnalyst tool. The rows are auto scaled and clustered. The clustering distance measure using Euclidean, and clustering algorithm using Ward. For abbreviations, m/z, retention time, and other details of the metabolites, refer to Supplementary Table 6.


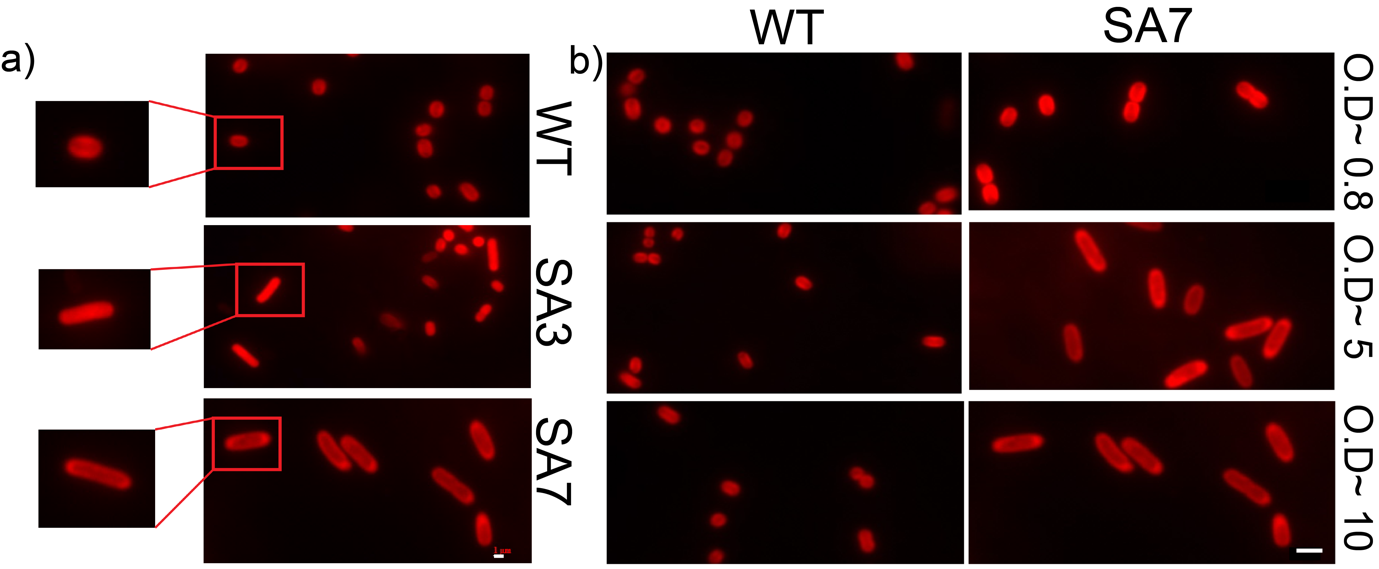


**Figure S14. Morphological analysis of succinate producing strains.** a) Morphological appearance of WT, SA3 and SA6 at 72 hours of growth by capturing the chlorophyll a fluorescence. Inset in the left panel showing the magnified image of the single cell. Scale bar is 1 μm. b) Morphological appearance of *S. elongatus* PCC 11801 SA7 compared to wildtype strain during different stages of growth. Cyanobacterial cells were harvested at different growth phases and fixed using 4% formaldehyde. The cells were imaged using fluorescence microscope. The OD_730_ of the cell culture is indicated. Scale bar represents 2 μm.

**Table S1:** List of recombinant strains of S. elongatus PCC 7942 and PCC 11801 constructed in the study.

| **Recombinant Strains** | **Description (△target site::genetic construct including the antibiotic marker)** |
| --- | --- |
| *S. elongatus* PCC 11801 SA1 | △DOP62_03525:: Sm^R^ P_rbcl400_ *OgdA* T_rbcl_ P_psbAIII_ *SsaD* T_rrnb_ |
| *S. elongatus* PCC 7942 SA1 | △Synpcc7942_2498:: Sm^R^ P_rbcl400_ *OgdA* T_rbcl_ P_psbAIII_ *SsaD* T_rrnb_ |
| *S. elongatus* PCC 11801 SA2 | △DOP62_03525:: Sm^R^ P_psbaI_ *OgdA* T _rbcl_ P_psbaIII_ *SsaD* T_rrnb_ |
| *S. elongatus* PCC 7942 SA2 | △Synpcc7942_2498:: Sm^R^ P_psbaI_ *OgdA* T_rbcl_ P_psbaIII_ *SsaD* T_rrnb_ |
| *S. elongatus* PCC 11801 SA3 | △DOP62_03525:: Sm^R^ P_psbaI_ *OgdA* T_rbcl_ P_psbaIII_ *SsaD* T_rrnb_ P_cpcb300_ *PEPC* T _rbcl_ |
| *S. elongatus* PCC 7942 SA3 | △Synpcc7942_2498:: Sm^R^ P_psbaI_ *OgdA* T_rbcl_ P_psbaIII_ *SsaD* T_rrnb_ P_cpcb300_ *PEPC* T_rbcl_ |
| *S. elongatus* PCC 11801 SA4 | △DOP62_03525:: Sm^R^ P_psbaI_ *OgdA* T_rbcl_ P_psbaIII_ *SsaD* T_rrnb_ P_cpcb300_ *PEPC* T_rbcl_ , ΔDOP62_11515 :: Gm^R^ |
| *S. elongatus* PCC 11801 SA5 | △DOP62_03525:: Sm^R^ P_psbaI_ *OgdA* T_rbcl_ P_psbaIII_ *SsaD* T_rrnb_ P_cpcb300_ *PEPC* T_rbcl_, ΔDOP62_03790 :: Kan^R^ |
| *S. elongatus* PCC 11801 SA6 | △DOP62_03525:: Sm^R^ P_psbaI_ *OgdA* T_rbcl_ P_psbaIII_ *SsaD* T_rrnb_ P_cpcb300_ *PEPC* , ΔDOP62_03790:: P_rbcl400_ *gltA* T_rbcl_ Kan^R^ |
| *S. elongatus* PCC 11801 SA7 | △DOP62_03525:: P_psbaI_ *OgdA* T_rbcl_ P_psbaIII_ *SsaD* T_rrnb_ P_cpcb300_ *PEPC* T_rbcl_, ΔDOP62_03790:: P_rbcl400_ *gltA* T_rbcl_ P_rbcl400_ *SBPase* T_rbcl_ Kan^R^ |
| *S. elongatus* PCC 11801 SA8 | △DOP62_03525:: Sm^R^ P_psbaI_ *OgdA* T_rbcl_ P_psbaIII_ *SsaD* T_rrnb_ P_cpcb300_ *PEPC* T_rbcl_, ΔDOP62_03790:: P_rbcl400_ *gltA* T_rbcl_ P_rbcl400_ *SBPase* T_rbcl_ Kan^R^, ΔDOP62_11515:: Gm^R^ |
| *S. elongatus* PCC 11801 SA9 | △DOP62_03525:: Sm^R^ P_psbaI_ *OgdA* T_rbcl_ P_psbaIII_ *SsaD* T_rrnb_ P_cpcb300_ *PEPC* T_rbcl_, ΔDOP62_03790:: P_rbcl400_ *gltA* T_rbcl_ P_rbcl400_ *SBPase* T_rbcl_ Kan^R^, ΔDOP62_11515:: Gm^R^ P_prbcl300M_ T_rbcl_ yjjP P_cpcb200_ yjjB T_lac_ |

**Table S2: List of plasmids constructed in the study.** The plasmids constructed here are derivatives of the plasmids pSYN1, pSYN6, pAM1619 and pAM3558. The pSYN1 and pSYN6 contain NSI homology arms of PCC 7942 and were not modified as the NSI sites of PCC 7942 and PCC 11801 share ~83% identity. The NSII homology arms of PCC 7942 of pAM1619(Mackey et al., 2007) were replaced with the upstream and downstream regions of *glgA* gene from PCC 11801. The *LuxCDE* genes were removed from the plasmid. The NSI homology arms of PCC 7942 in plasmid pAM3558 (Chen et al., 2008) were replaced with upstream and downstream homology arms of *sdhB* gene from PCC 11801.

| **Parent Plasmid** | **Derived Plasmids** | **Description (antibiotic marker; target site; elements of the genetic construct)** |
| --- | --- | --- |
| pSYN1 | pSYN1_A | Spec^R^; NSI targeting; P_rbcl400_ *OgdA* T_rbcl_ P_psbAIII_ *SsaD* T_rrnb_ |
| pSYN6 | pSYN6_B | Spec^R^; NSI targeting; P_psbAI_ *OgdA* T_rbcl_ P_psbAIII_ *SsaD* T_rrnb_ |
| pSYN6 | pSYN6_C | Spec^R^; NSI targeting; P_psbaI_ *OgdA* T_rbcl_ P_psbaIII_ *SsaD* T_rrnb_ P_cpcb300_ *PEPC* T_rbcl_ |
| pAM1619 | pAM1619_D | Kan^R^; *glgA* targeting; upstream and downstream region. |
| pAM1619_D | pAM1619_E | Kan^R^; *glgA* targeting; upstream and downstream region; P_rbcl400_ *gltA* T_rbcl_. |
| pAM1619 | pAM1619_F | Kan^R^; *glgA* targeting; upstream and downstream region; P_rbcl400_ *gltA* T_rbcl_ P_rbcl400_ *SBPase* T_rbcl_. |
| pAM3558 | pAM3558_G | Genta^R^; *sdhB* targeting; upstream and downstream region. |
| pAM3558 | pAM3558_H | Genta^R^; *sdhB* targeting; upstream and downstream region; P_prbcl300M_ T_rbcl_ yjjP P_cpcb200_ yjjB T_lac_. |

**Table S3. The sequence of strain construction.** Each recombinant strain was constructed by transforming the WT or a recombinant strain with the specified plasmid with either natural transformation or triparental conjugation. Complete chromosomal segregation was achieved before proceeding with the use of the recombinant strain for further transformation.

| Sequence of Strain Construction | Transformation Method |
| --- | --- |
| PCC 11801 WT + pSYN1_A → PCC 11801 SA1 | Natural Transformation |
| PCC 11801 WT + pSYN6_B → PCC 11801 SA2 | Natural Transformation |
| PCC 11801 WT + pSYN6_C → PCC 11801 SA3 | Natural Transformation |
| PCC 7942 WT + pSYN1_A → PCC 7942 SA1 | Natural Transformation |
| PCC 7942 WT + pSYN6_B → PCC 7942 SA2 | Natural Transformation |
| PCC 7942 WT + pSYN6_C → PCC 7942 SA3 | Natural Transformation |
| PCC 11801 SA3 + PAM3558_G → PCC 11801 SA4 | Natural Transformation |
| PCC 11801 SA3 + pAM1619_D → PCC 11801 SA5 | Natural Transformation |
| PCC 11801 SA3 + pAM1619_E → PCC 11801 SA6 | Natural Transformation |
| PCC 11801 SA3 + pAM1619_F → PCC 11801 SA7 | Natural Transformation |
| PCC 11801 SA7 + pAM3558_G → PCC 11801 SA8 | Triparental conjugation |
| PCC 11801 SA7 + pAM3558_H → PCC 11801 SA9 | Triparental conjugation |

**Table S4. List of primers used for preparing the genetic constructs, plasmids and confirmation PCR.**

| **Primers** | **Sequences** | **Used for** |
| --- | --- | --- |
| **For construction of plasmid pSYN1_A** | | |
| IITB 1 | GACATAAAGCTTTGATCAGTGATGGAAAAAGCACTGTAATTC | P_rbcl400_ |
| IITB 2 | CAACGAATCAGCAGCTCGGCGGTGTTCATGTCGTCTCTCCCTAGAGATATGTCAGA |  |
| IITB 3 | TCTGACATATCTCTAGGGAGAGACGACATGAACACCGCCGAGCTGCTGATTC | *OgdA* |
| IITB 4 | GAGTACAACTCCGACAATCCAAACACCGGTTTTACATACGACAGATCAGGTCGCCGCTCT |  |
| IITB 5 | AAGAGCGGCGACCTGATCTGTCGTATGTAAAACCGGTGTTTGGATTGTCGGAGTTGTACTC | T_rbcl_ |
| IITB 6 | CTACTGCAACTTCCGGCGCGATCCCGGAACGAAGTTGAACATCAGTAAGCAGT |  |
| IITB 7 | CCCACTGCTTACTGATGTTCAACTTCGTTCCGGGATCGCGCCGGAAGT | P_psbAIII_ |
| IITB 8 | GCGGAATAAGTTACTGTCGTTAAGTTTCATGATGTTTTGAGTCCAGTGAATTTTTATG |  |
| IITB 9 | CATAAAAATTCACTGGACTCAAAACATCATGAAACTTAACGACAGTAACTTATTCCGC | *SsaD* |
| IITB 10 | GACCACCGCGCTACTGCCGCCAGGCA TTAAAGACCGATGCACATATATTTGATTTC |  |
| IITB 11 | CAAATATATGTGCATCGGTCTTTAATGCCTGGCGGCAGTAGCGCGGTGGTC | T_rrnb_ |
| IITB 12 | GACTAGCTCGAGAAGGCCCAGTCTTTCGACTGAGCC |  |
| **For construction of plasmid pSYN6_B** | | |
| IITB 13 | GCCATAAAGCTTATGAACACCGCCGAGCTGCTGATTC | *OgdA* |
| IITB 4-IITB 12 | Same as above | Rest of the construct |
| **For construction of plasmid pSYN6_C** | | |
| IITB 14 | GATTAC CTCGAG TAGTAATGA CACAATTTTTAGCGAATCTTGTGGC | P_cbcb300_ |
| IITB 15 | A GTTCGAGCGTTCTGGCAGTAATTCATTCAACCAGTCTCCTGTTCTCGACTTT |  |
| IITB 16 | AAAGTCGAGAACAGGAGACTGGTTGAATGAATTACTGCCAGAACGCTCGAACTG | *PEPC* |
| IITB 17 | GTACAACTCCGACAATCCAAACACCGGTTTCAACCTGTATTGCGCATGCCAGCCGCAATG |  |
| IITB 18 | CATTGCGGCTGGCATGCGCAATACAGGTTGAAACCGGTGTTTGGATTGTCGGAGTTGTAC | T_rbcl_ |
| IITB 19 | GCCGGATATCCGAAGTTGAACATCAGTAAGCAGTG |  |
| **For construction of plasmid pSYN6_D** | |  |
| IITB 20 | GTCCTCCTCAGCGGTCGGAGGAGGCAATCGCTCATG | glgA downstream |
| IITB 21 | GTTCCGGCATGCTCAGGGATCGCGACGAACAATGAGC |  |
| IITB 22 | GCCTCAGTACTGATCTCAAGACTGGCACCCTCGTTG | glgA upstream |
| IITB 23 | GCTCTGAATTCGCAGACCTCAGGGGGCAACGCCCTCC |  |
| **For construction of plasmid pSYN6_E** | |  |
| IITB 24 | CCGGA GAATTC TGATCAGTGATGGAAAAAGCACTGTAATTC | P_rbcl400_ |
| IITB 25 | GGCCAGGCCGAAACTCGCTGACGGCAGTCATGTCGTCTCTCCCTAGAGATATGTCAGAT |  |
| IITB 26 | ATATCTGACATATCTCTAGGGAGAGACGACATGACTGCCGTCAGCGAGTTTCGGC | *gltA* |
| IITB 27 | GTACAACTCCGACAATCCAAACACCGGTTTCAATCAGATTCGATCGCCAAATCCCGATCG |  |
| IITB 28 | TTGGCGATCGAATCTGATTGA AACCGGTGTTTGGATTGTCGGAGTTGTAC | T_rbcl_ |
| IITB 29 | GTCGC GGTACC CGAAGTTGAACATCAGTAAGCAGTG |  |
| **For construction of plasmid pSYN6_F** | |  |
| IITB 30 | CCGGA GGTACC TGATCAGTGATGGAAAAAGCACTGTAATTC | P_rbcl400_ |
| IITB 31 | CTCGGAAGTGGTGGATTGAGCCATGTCGTCTCTCCCTAGAGATATGTCAG |  |
| IITB 32 | ATATCTGACATATCTCTAGGGAGAGACGACATGGCTCAATCCACCACTTCCGAGAC | *SBPase* |
| IITB 33 | GTACAACTCCGACAATCCAAACACCGGTT AGCCAAGCAGGCTTCGACAAAGTCC |  |
| IITB 34 | GGACTTTGTCGAAGCCTGCTTGGCTAACCGGTGTTTGGATTGTCGGAGTTGTAC | T_rbcl_ |
| IITB 35 | GTCGC GCTAGC CGAAGTTGAACATCAGTAAGCAGTG |  |
| **For construction of plasmid pSYN6_G** | | |
| IITB 36 | CGAAACGCGCGAGGCAGGATCG CGCGATCGGACAGCTTCTAGCGC | sdhB upstream |
| IITB 37 | GACCCCGGATGAAGTTCGAGCATGC GGGAGCGATCGCCGGTAATACAGTC |  |
| IITB 38 | CCGGGTACCGAGCTCGAATT CTAAACTTTTTCGCCACTGGCTACTC | sdhB downstream |
| IITB 39 | GATGCTTTTCTGTGACTGGTGAGT GGCTCAGCCCCCAGACGGGTCAACCG |  |
| **For construction of plasmid pSYN6_H** | |  |
| IITB 40 | CAAGCATACTAGAGGATCGGCGGCC GGATCC AGACGGGAGGCGGCTTTTGCCATG | P_rbcl300_^M^ |
| IITB 41 | CTAACAGAAATTCGATCACACCCAT GTCGTCTCTCCCTAGAGATATGTCAGATATG |  |
| IITB 42 | CTGACATATCTCTAGGGAGAGACGAC ATGCAAACTGAGCAACAGCGAGCCG | *yjjB* |
| IITB 43 | GACCACCGCGCTACTGCCGCCAGGCA CACCCATCCGCGTAGCCCCCAAATCG |  |
| IITB 44 | GGTTGTACCGCAAGCGCCCTCGCGT TGCCTGGCGGCAGTAGCGCGGTGGTCCC | T_rrnB_ |
| IITB 45 | ACGAGAGGGGCCTGTCAGGCAATTG AAGGCCCAGTCTTTCGACTG |  |
| IITB 46 | GTCGAGAACAGGAGACTGGTTGA ATGCAAACTGAGCAACAGCGAGCCG | *yjjP* |
| IITB 47 | GCAGCGTATCAGGCAATTTTTATAAT CACCCATCCGCGTAGCCCCCAA |  |
| IITB 48 | CGAAAGGCTCAGTCGAAAGACTGG CAATTGCCTGACAGGCCCCTCTCG | P_cpcb200_ |
| IITB 49 | CGGCTCGCTGTTGCTCAGTTTGCAT TCAACCAGTCTCCTGTTCTCGACTT |  |
| IITB 50 | GATTTGGGGGCTACGCGGATGGGTG ATTATAAAAATTGCCTGATACGCTGC | T_Lac_ |
| IITB 51 | CTGCTCCAGAAGCTCGAAATTC GAGCTC ACTGGGCATTTCATGCGCTG |  |
|  | Same as IITB 36 and 37 | sdhB upstream |
| IITB 54 | GAAATGCCCAGGAGCTCGAATT CTAAACTTTTTCGCCACTGGCTACTC | sdhB downstream |
|  | Same as IITB 39 |  |
| IITB 56 | GACATTGCTACCGAAGTCGCTCGTAGCCAAGCAG | Confirmation primers for NSI |
| IITB 57 | CAGATCAATGCCCGTGGTTTGTTTCAGCTTCTC |  |
| IITB 58 | CTAGGCCTCCTGCAAGACATCGATC | *glgA* |
| IITB 59 | ATGCGGATTCTGTTCGTAGCTGCCGAATGTG |  |
| IITB 60 | *TTAGCTGTTTTGCCAGCGGGCCTG* | *sdhB* |
| IITB 61 | ATGCCCAGTGCCGTTCTCGACCTGAACTTT |  |
| IITB 62 | ACCTCGCATATGAAAGTCGCAGTCCTCGGCGCT | *mdh* |
| IITB 63 | AACTAGCTCGAGATTACTTATTAACGAACTCTTCGCCCAGGCG |  |

**Table S5. List of primers used for the reverse transcriptase-PCR study of selected genes.** Each primer set results in an amplicon of ~ 200bp.

| Primer | Sequence | Used for | Organism |
| --- | --- | --- | --- |
| SC1 | CCGGCGTTTTCACCTCAAAG | *zwf* | PCC 11801 |
| SC2 | GATGGTCGATAACTGGCGCT |  |  |
| SC3 | GCCTTATCCAACGGGCAGAT | *Pyruvate dehydrogenase* | PCC 11801 |
| SC4 | TGAACACTCCCAACGACTGG |  |  |
| SC5 | GATCGCCTTCGACCATGACT | *SdhB* | PCC 11801 |
| SC6 | GCACTGCGATCGACGACTAA |  |  |
| SC7 | GTCGAGACGATGAAGCGGAT | *Malic Enzyme* | PCC 11801 |
| SC8 | CGTACCTGCGACAGGGATTT |  |  |
| SC9 | CTTCCATCTGCTCGATCGCT | *Pyruvate Kinase* | PCC 11801 |
| SC10 | ATCTGTCCGTCAAAGCCCTG |  |  |
| SC11 | GGAAATCCACAAGGCCCAGA | *PEPC* | PCC 11801 |
| SC12 | AGGCAGGGCATATTTCGAGG |  |  |
| SC13 | GATCCCTACGCTGTAGTCGC | *Citrate synthase* | PCC 11801 |
| SC14 | CTGCCCGTGGATCTTTGACT |  |  |
| SC15 | GCGGGCTATGTCCTCTATGG | *SBPase* | PCC 11801 |
| SC16 | TCCCGGATGTACTGGCGATA |  |  |
| SC17 | CGCCAAATCGCTGCTCAAAT | *RBCL* | PCC 11801 |
| SC18 | AGCCAGCAACGCGGATATAG |  |  |
| SC19 | ACGTCGATTTCTACTCCGGC | *Citrate synthase* | PCC 7942 |
| SC20 | ATTCGATCGCCAAATCCCGA |  |  |
| SC21 | GCAAAGCTTTAGCCCCGAAG | *SBPase* | PCC 7942 |
| SC22 | GCCACTCTGCCGAAAAACTG |  |  |
| SC23 | CGCGAGAACACTGAGGACAT | *Isocitrate dehydrogenase* | PCC 11801 |
| SC24 | GATCGCACGACGAATCAAGC |  |  |
| SC25 | CTGGATGGTGCCCCGTTAAT | *OgdA* | PCC 7942 |
| SC26 | TCCTGCTGTGCGATCTTGAA |  |  |
| SC27 | GCCTGATGACCCTCGAACAG | *SsaD* | PCC 7942 |
| SC28 | AAGTTCCACGGCGTGATAGC |  |  |
| SC29 | TCTTCCGCATGGTGATCTCG | *PEPC* | PCC 7942 |
| SC30 | AAGCGACCGTGGTTGGTAAT |  |  |
| SC31 | GGTGATACCGCAGGGTTCAA | *GLGA* | PCC 11801 |
| SC32 | TCGCTTCCTGTCTTACAGCG |  |  |
| SC33 | AGCCACACTGGGACTGAGAC | *16S rRNA* | PCC 11801 |
| SC34 | TTCCCTGAGAAAAGGGGTTT |  |  |

**Table S6. List of metabolites studied in this study.**

|  | Abbreviation | Compound Name | Formula | m/z Da | RT(min) | KEGG ID |
| --- | --- | --- | --- | --- | --- | --- |
| 1 | GLY | Glycine | C_2_H_5_NO_2_ | 74.03 | 1.1 | C00037 |
| 2 | ­­­­­­ALA | Alanine | C_3_H_7_NO_2_ | 88.05 | 1.2 | C00041 |
| 3 | GABA | Gamma-aminobutyric acid | C_4_H_9_NO_2_ | 102.06 | 6.0 | C00334 |
| 4 | SER | Serine | C_3_H_7_NO_3_ | 104.04 | 1.2 | C00065 |
| 5 | ---- | Glyceric acid | C_3_H_6_O_4_ | 105.02 | 7.4 | C00258 |
| 6 | VAL | Valine | C_5_H_11_NO_2_ | 116.03 | 1.5 | C00183 |
| 7 | ---- | Succinate | C_4_H_6_O_4_ | 117.02 | 13.3 | C00042 |
| 8 | ---- | Pyroglutamic acid | C_5_H_7_NO_3_ | 128.04 | 6.0 | C01879 |
| 9 | LEU | Leucine | C_6_H_13_NO_2_ | 130.08 | 2.6 | C00123 |
| 10 | ASP | Aspartate | C_4_H_7_NO_4_ | 132.03 | 6.0 | C00049 |
| 11 | GLN | Glutamine | C_5_H_10_N_2_O_3_ | 145.09 | 1.2 | C00064 |
| 12 | GLU | Glutamate | C_5_H_9_NO_4_ | 146.05 | 5.7 | C00025 |
| 13 | PEP | Phosphoenolpyruvate | C_3_H_5_O_6_P | 166.98 | 15.0 | C00074 |
| 14 | --- | Glycerol-3-phosphate | C_3_H_9_O_6_P | 171.01 | 11.5 | C00093 |
| 15 | CIR | Citrulline | C_6_H_13_N_3_O_3_ | 174.11 | 1.3 | C00327 |
| 16 | NCD | N-carbamoyl aspartate | C_5_H_8_N_2_O_5_ | 175.04 | 13.2 | C00438 |
| 17 | ---- | 2-isopropylmalate | C_7_H_12_O_5_ | 175.04 | 15.1 | C02504 |
| 18 | 3PGA | 3-phosphoglycerate | C_3_H_7_O_7_P | 184.99 | 14.7 | C00197 |
| 19 | N-Acetyl-GLU | N-acetylglutamate | C_7_H_11_NO_5_ | 188.06 | 13.6 | C00624 |
| 20 | G6P | Glucose-6-phosphate | C_6_H_13_O_9_P | 259.02 | 10.6 | C00668 |
| 21 | F6P | Fructose-6-phosphate | C_6_H_13_O_9_P | 259.02 | 10.9 | C00085 |
| 22 | 6PG | 2-Keto-3-deoxy-6-phosphogluconate | C_6_H_13_O_10_P | 274.98 | 14.7 | C04442 |
| 23 | S7P | Sedoheptulose 1,7-bisphosphate | C_7_H_15_O_10_P | 289.03 | 10.9 | C05382 |
| 24 | GlcNAc-6P | N-acetylglucosamine-6-phosphate | C_8_H_16_NO_9_P | 300.03 | 11.7 | C00357 |
| 25 | RuBP | Ribulose 1,5 bisphosphate | C_5_H_12_O_11_P_2_ | 308.98 | 15.2 | C01182 |
| 26 | Deoxy-TMP | deoxy-thymidine monophosphate | C_10_H_15_N_2_O_8_P | 321.07 | 12.9 | C00364 |
| 27 | CMP | Cytidine-5'-monophosphate | C_9_H_14_N_3_O_8_P | 322.05 | 11.6 | C00055 |
| 28 | UMP | Uridine monophosphate | C_9_H_13_N_2_O_9_P | 323.03 | 11.9 | C00105 |
| 29 | FBP | Fructose-1,6 bisphosphate | C_6_H_14_O_12_P_2_ | 338.96 | 15.1 | C05378 |
| 30 | SUC | Sucrose | C_12_H_22_O_11_ | 341.11 | 1.5 | C00089 |
| 31 | AMP | Adenosine 5'-monophosphate | C_10_H_14_N_5_O_7_P | 346.06 | 13.7 | C00020 |
| 32 | IMP | Inosine 5'-monophosphate | C_10_H_13_N_4_O_8_P | 347.05 | 12.1 | C00130 |
| 33 | SBP | Sedoheptulose 1,7 bisphosphate | C_7_H_16_O_13_P_2_ | 369.01 | 15.2 | C00447 |
| 34 | GMP | Guanosine monophosphate | C_10_H_14_N_5_O_8_P | 362.02 | 12.4 | C00144 |
| 35 | XMP | Xanthosine monophosphate | C_10_H_13_N_4_O_9_P | 363.04 | 14.8 | C00655 |
| 36 | CDP | Cytidine diphosphate | C_9_H_15_N_3_O_11_P_2_ | 402.04 | 13.7 | C00112 |
| 37 | UDP | Uridine diphosphate | C_9_H_14_N_2_O_12_P_2_ | 403.00 | 14.8 | C00015 |
| 38 | S-6-P | Sucrose-6-phosphate | C_12_H_23_O_14_P | 421.08 | 10.5 | C16688 |
| 39 | ADP | Adenosine diphosphate | C_10_H_15_N_5_O_10_P_2_ | 426.02 | 14.9 | C00008 |
| 40 | GDP | Guanosine diphosphate | C_10_H_15_N_5_O_11_P_2_ | 442.02 | 14.7 | C01228 |
| 41 | ATP | Adenosine triphosphate | C_10_H_16_N_5_O_13_P_3_ | 505.99 | 15.3 | C00002 |
| 42 | GTP | Guanosine triphosphate | C_10_H_16_N_5_O_14_P_3_ | 521.99 | 15.2 | C00044 |
| 43 | UDX | UDP-xylose | C_14_H_22_N_2_O_16_P_2_ | 535.04 | 13.1 | C00190 |
| 44 | dTDP-Rham | deoxy-TDP-rhamnose | C_16_H_26_N_2_O_15_P_2_ | 547.08 | 14.1 | C03319 |
| 45 | UPG | UDP-glucose | C_15_H_24_N_2_O_17_P_2_ | 565.05 | 13.2 | C00029 |
| 46 | UGA | UDP-glucuronate | C_15_H_22_N_2_O_18_P_2_ | 579.03 | 15.0 | C00167 |
| 47 | ADPG | ADP-glucose | C_16_H_25_N_5_O_15_P_2_ | 588.08 | 14.1 | C00498 |
| 48 | GFB | GDP-fucose | C_16_H_25_N_5_O_15_P_2_ | 588.08 | 13.6 | C00325 |
| 49 | GDD | GDP-mannose | C_16_H_25_N_5_O_16_P_2_ | 604.07 | 13.1 | C00096 |
| 50 | UD1 | UDP-N-acetyl-glucosamine | C_17_H_27_N_3_O_17_P_2_ | 606.08 | 13.1 | C00043 |
| 51 | GDS | Oxidized glutathione | C_20_H_32_N_6_O_12_S_2_ | 611.15 | 12.4 | C00127 |
| 52 | CMP-Neu5Ac | Cytidine monophosphate N-acetylneuraminic acid | C_20_H_31_N_4_O_16_P | 613.15 | 12.5 | C00128 |
| 53 | ADP-ribose CP | ADP ribose 1,2 cyclic phosphate | C_15_H_22_N_5_O_16_P_3_ | 620.02 | 15.0 | C19851 |
| 54 | NAD | Nicotinamide adenine dinucleotide | C_21_H_28_N_7_O_14_P_2_ | 663.11 | 10.3 | C00003 |
| 55 | UDP-MurNAc | UDP-N-acetylmuraminate | C_20_H_31_N_3_O_19_P_2_ | 678.10 | 15.1 | C01050 |
| 56 | NADP | Nicotinamide adenine dinucleotide phosphate | C_21_H_29_N_7_O_17_P_3_ | 743.08 | 14.9 | C00003 |
| 57 | FAD | Flavin adenine dinucleotide | C_27_H_33_N_9_O_15_P_2_ | 784.15 | 15.3 | C00016 |
| 58 | Ac-CoA | Acetyl Coenzyme A | C_23_H_38_N_7_O_17_P_3_S | 808.13 | 15.6 | C00024 |
| 59 | 3-oxohex-CoA | 3-oxohexanoyl coenzyme A | C_27_H_44_N_7_O_18_P_3_S | 878.18 | 15.5 | C05269 |
| 60 | --- | Glycogen | C_24_H_42_O_21_ | Not detected | | COO182 |

**Table S7. Sequence of promoters used in the study.** All the promoters used in the study are constitutive native promoters amplified from the genome of PCC 7942. Prbc400M is a synthetic promoter created by random mutagenesis (Unpublished work, A. Sengupta et al., 2020).

| **Promoter** | **Sequence** |
| --- | --- |
| P_psbAI_ | GCTGGCTATTTAGCGTCTTCTAATCCAGTGTAGACAGTAGTTTTGGC  TCCGTTGAGCACTGTAGCCTTGGGCGATCGCTCTAAACATTACATA  AATTCACAAAGTTTTCGTTACATAAAAATAGTGTCTACTTAGCTAA  AAATTAAGGGTTTTTTACACCTTTTTGACAGTTAATCTCCTAGCCTA  AAAAGCAAGAGTTTTTAACTAAGACTCTTGCCCTTTACAACCTCGAAG |
| P_psbAIII_ | TTCCGGGATCGCGCCGGAAGTTGCAGTAGGTGCAGCGATTGAAGCA  CTCGTAGGTGGGAACGAGGGTATAGGCCGGACTGTAGGTGATCACA  GCTGATTGTTGGGGCGATCGCCAAGTTTGATCTGAGAAGTGCTTCAC  AATTCGCAAAGTTTTGTTATTATTAGCTCATAAGGCATTACGCCTTAG  ACATACCATAAAAATTCACTGGACTCAAAACATC |
| P_rbc400_^(Sengupta et al., 2019)^ | TGATCAGTGATGGAAAAAGCACTGTAATTCCCTTGGTTTTTGGCTG  AAAGTTTCGGACTCAGTAGACCTAAGTACAGAGTGATGTCAACGC  CTTCAAGCTAGACGGGAGGCGGCTTTTGCCATGGTTCAGCGATCGC  TCCTCATCTTCAATAAGCAGGGCATGAGCCAGCGTTAAGCAAATCA  AATCAAATCTCGCTTCTGGGCTTCAATAAATGGTTCCGATTGATGAT  AGGTTGATTCATGAGGAATCTAAGGCTTAATTCTCCACAAAAGAAT  TAAGCGTCCGTCGCAACGGAATGCTCCGCTGGACTTGCGCTGTGGG  ACTGCAGCTTTACAGGCTCCCCCTGCCAGAAATCCTGAATCGTCGAG  CATATCTGACATATCTCTAGGGAGAGACGAC |
| P_rbc300_^M^ | AGACGGGAGGCGGCTTTTGCCATGGTTCAGCGATCGCTCCACATCT  TCAATAAGCAGGGCATGAGCCAGCGTTAAGCAAATCAAATCAAAT  CTCGCTTCAGGGCTTCAATAAATGGTTCCGATTGATGATAGGTTGA  TTCATGAGGAATCTAAGGCTTAATTCTCCACAAAAGAATTAAGCGT  CCGTCGCAACGGAATGCTCCGCTGGACTTGCGCTGTGGGACTGCAG  CTTTACAGGCTCCCCCTGCCAGAAATCCTGAATCGTCGAGCATATCT  GACATATCTCTAGGGAGAGACGAC |
| P_cpcb300_(Sengupta et al., 2019) | CACAATTTTTAGCGAATCTTGTGGCCGCGATCGTTGTATAAGAATG  CCAGGCAACTGGATAAGGTTCACTAATCGTTGCTAAGCGACAGTGA  ACTGCGCCAATTGCCTGACAGGCCCCTCTCGTTTAACAAACGATTTA  ATGTAAATCATTGTTAAGAGTCTCTCACAATCGAGAGTTTTCTTGAA  GAATGATGGGGACGGTTCAGGTGCAGGGTTTCCCTGCTAGAGAATG  CGAAAAAACCGCGTTCTCGTTTTAGGAATCGAGAGTCAATAAAAGT  CGAGAACAGGAGACTGGTTGA |
| P_cpcb200_(Sengupta et al., 2019) | CAATTGCCTGACAGGCCCCTCTCGTTTAACAAACGATTTAATGTAA  ATCATTGTTAAGAGTCTCTCACAATCGAGAGTTTTCTTGAAGAATG  ATGGGGACGGTTCAGGTGCAGGGTTTCCCTGCTAGAGAATGCGAA  AAAACCGCGTTCTCGTTTTAGGAATCGAGAGTCAATAAAAGTCGA  GAACAGGAGACTGGTTGA |

**References.**

1. Chen, Y., Holtman C.K., Magnuson, R.D., Magnuson R.D., Youderian, P.A., Youderian P.A., Golden, S.S., and Golden, S.S. (2008). The complete sequence and functional analysis of pANL, the large plasmid of the unicellular freshwater cyanobacterium Synechococcus elongatus PCC 7942. Plasmid *59*, 176-192.
2. Mackey, S.R., JL, D., EM, C., and Golden, S.S. (2007). Detection of rhythmic bioluminescence from luciferase reporters in cyanobacteria. Methods in Molecular Biology *362*, 115-129.
3. Sengupta, A., Sunder, A.V., Sohoni, S.V., and Wangikar, P.P. (2019). Fine-Tuning Native Promoters of Synechococcus elongatus PCC 7942 To Develop a Synthetic Toolbox for Heterologous Protein Expression. ACS Synthetic Biology.
